# Purification, Cloning, Characterization, and N-Glycosylation Analysis of a Novel β-Fructosidase from *Aspergillus oryzae* FS4 Synthesizing Levan- and Neolevan-Type Fructooligosaccharides

**DOI:** 10.1371/journal.pone.0114793

**Published:** 2014-12-12

**Authors:** Li Xu, Dongxue Wang, Lili Lu, Lan Jin, Jiawei Liu, Deyong Song, Zhongwu Guo, Min Xiao

**Affiliations:** State Key Lab of Microbial Technology and National Glycoengineering Research Center, Shandong University, Jinan, PR China; University of Delhi, India

## Abstract

β-Fructosidases are a widespread group of enzymes that catalyze the hydrolysis of terminal fructosyl units from various substrates. These enzymes also exhibit transglycosylation activity when they function with high concentrations of sucrose, which is used to synthesize fructooligosaccharides (FOS) in the food industry. A β-fructosidase (BfrA) with high transglycosylation activity was purified from *Aspergillus oryzae* FS4 as a monomeric glycoprotein. Compared with the most extensively studied *Aspergillus* spp. fructosidases that synthesize inulin-type β-(2-1)-linked FOS, BfrA has unique transfructosylating property of synthesizing levan- and neolevan-type β-(2-6)-linked FOS. The coding sequence (*bfrA*FS4, 1.86 kb) of BfrA was amplified and expressed in *Escherichia coli* and *Pichia pastoris*. Both native and recombinant proteins showed transfructosylation and hydrolyzation activities with broad substrate specificity. These proteins could hydrolyze the following linkages: Glc α-1, 2-β Fru; Glc α-1, 3-α Fru; and Glc α-1, 5-β Fru. Compared with the unglycosylated *E. coli*-expressed BfrA (E.BfrA), the N-glycosylated native (N.BfrA) and the *P. pastoris*-expressed BfrA (P.BfrA) were highly stable at a wide pH range (pH 4 to 11), and significantly more thermostable at temperatures up to 50°C with a maximum activity at 55°C. Using sucrose as substrate, the *Km* and *k_cat_* values for total activity were 37.19±5.28 mM and 1.0016±0.039×10^4^ s^−1^ for N.BfrA. Moreover, 10 of 13 putative N-glycosylation sites were glycosylated on N.BfrA, and N-glycosylation was essential for enzyme thermal stability and optima activity. Thus, BfrA has demonstrated as a well-characterized *A. oryzae* fructosidase with unique transfructosylating capability of synthesizing levan- and neolevan-type FOS.

## Introduction

Fructooligosaccharides (FOS) are widely used as a bioactive ingredient in functional foods because of their prebiotic properties. FOS stimulate the growth of potential health-promoting bacteria, such as bifidobacteria and lactobacilli [1] as well as improve calcium and magnesium absorption in adolescents and in postmenopausal women [2]. FOS, generally considered as functional foods, are mainly known as inulin-type with β-(2-1) fructosyl linkage [3], [4]. Levan- and neolevan-type FOS that contain fructose units linked by β-(2-6) linkages exhibit increased prebiotic activity compared with the usual inulin-type FOS [3], [5]. Therefore, enzymes that produce different types of FOS have attracted much attention. β-Fructosidase (invertase, β-fructofuranosidases, EC 3.2.1.26) is the main enzyme for the commercial production of FOS. Based on overall amino acid sequence similarities, β-fructosidase belongs to the glycosyl hydrolase family 32 (GH32) and shares a common three-dimensional (3-D) structure with other GH32 members [6]. β-Fructosidase catalyzes the hydrolysis of non-reducing termini of various substrates such as sucrose, raffinose, inulin, and levan. Several microbial β-fructosidases could also catalyze the synthesis of short-chain FOS, in which one to three fructosyl moieties are linked to the sucrose by different glycosidic bonds depending on the enzyme source. Given the high production of FOS, the industrial application of these FOS largely relies on fungal enzymes from *Aspergillus* spp. [1], and *Aspergillus oryzae* has been considered as an attractive source of enzymes for this process [7], [8]. Although the development of novel FOS, such as levan- or neolevan-type β-(2-6) structure [3], [5], has been attracting considerable interest, only inulin-type [β-(2-1)-linked] FOS are reported to be produced by *Aspergillus* spp. using sucrose as substrate [1], [9]. The novel enzyme discovered from *Aspergillus* spp. that synthesizes β-(2-6)-linked FOS may have a great potential for production of FOS in the food industry.

Enzymes used in the food industry are preferably thermostable and pH tolerant. Enzymes derived from microorganisms are more thermostable than those derived from plants. Accordingly, much attention has been paid to the exploitation of β-fructosidase from various microbial sources and improvement of the enzyme thermostability for industrial applications. Glycosylation is one of the most important post-translational modifications, and glycans on a secreted protein modulate its properties, such as protein folding, stability, and even function. The effect of glycosylation on thermostability has been investigated for various proteins. Lige et al. demonstrated that the removal of one of the N-glycosylation recognition sites on a peroxidase from peanut significantly reduced enzyme thermostability [10], which was also observed for rice a-amylase1A [11]. In addition, glycosylated hAQP10 exhibits a remarkably higher thermostability than its non-glycosylated counterpart [12]. Clark et al. [13] successfully enhanced the thermostability of this enzyme by adding N-glycosylation recognition sites on recombinant barley α-glucosidase molecule. In fungi, extracellular β-fructosidases commonly contain putative N-linked glycosylation sites [14], [15], [16], [17], [18], [19], [20], [21]. It remains unknown whether any of these putative N-glycosylation sites are glycosylated. Whether the absence of N-glycosylation could affect the functions of these enzymes has not been studied, either.

The purification, cloning, heterologous expression, and characterization of a β-fructosidase (BfrA) from *A. oryzae* FS4 were described in the present study. In addition to the broad substrate-hydrolytic activity, this enzyme displayed high transfructosylating activity with a fructooligosaccharide yield of approximately 56%. Compared with β-(2-1) glycosidic-bond FOS produced by most *Aspergillus* spp. fructosidases, the FOS synthesized by this enzyme were levan and neolevan types with β-(2-6) glycosidic bonds. The native and the recombinant BfrA enzymes were characterized. Moreover, the putative N-glycosylation sites of the enzyme were analyzed by mass spectrometry (MS), and the glycan was confirmed to contribute to enzyme optimal activity and thermostability. The successful cloning, expression, and characterization of this enzyme resulted in the further understanding of the mechanisms that determine the formation of either (2-1) or (2-6) glycosidic linkages of FOS, which will lead to the structural and/or functional studies on more GH32 family proteins.

## Methods and Materials

### Materials and strains

The glucose assay kit was obtained from Biosino Bio-Technology and Science Inc. (China), and the enzymatic deglycosylation kit from Prozyme (USA). Turanose, leucrose, palatinose, and raffinose were obtained from Sigma-Aldrich (USA). The other chemicals were of analytical grade and are commercially available.

*A. oryzae* FS4 (CGMCC No. 9087) was isolated and stored on potato dextrose agar slants at 4°C and cultured at 28°C for 48 h in a fermentation medium that contained 2% sucrose, 3% yeast extract, and 0.5% carboxymethyl cellulose. *E. coli* DH5a, *E. coli* BL21, and *Pichia pastoris* KM71 were cultured based on the protocols from the pET System Manual (Novagen, Germany) and the *Pichia* Expression Kit (Invitrogen, USA).

### Purification of native BfrA (N.BfrA) from *A. oryzae* FS4

All purification steps were conducted at 4°C, unless otherwise mentioned. The mycelia were harvested by a filter and ground in liquid nitrogen after they were lyophilized by vacuum freeze drying in 1∶16 (w/w) ratio of potassium phosphate buffer (KPB, 50 mM, pH 7.0). The resulting crude enzyme solution was concentrated by ammonium sulfate precipitation (50% to 85% saturation) and then desalinated. The enzyme solution was then sequentially applied to a DEAE–Sepharose Fast Flow column (1.6×15 cm, GE Healthcare, USA) followed by a Superdex-200 column (1.5×87 cm, GE Healthcare, USA). Fractions with enzymatic activity were dialyzed, concentrated, and further purified by native gradient PAGE (5% to 15%). For the final native PAGE purification, the location of the protein band was determined by Coomassie blue staining and the corresponding band was excised from the nonstained lane. The protein fractions were separated from gel slices for activity assay and further electrophoretic analysis.

### Enzymatic activity assay and determination of kinetic parameters

The β-fructosidase activity was measured in a 50 µL volume that contained 36 ng enzyme and 50 mM sucrose in KPB buffer. The reaction was performed at 55°C for 20 min and then stopped by heating in boiled water for 1 min. The amount of released glucose was measured by the glucose assay kit. One unit of enzymatic activity (U) was defined as the amount of enzyme required to liberate 1 µmol of glucose per minute under the assay conditions.

The kinetic parameters of total enzyme activity (glucose release from sucrose) [22] were determined at 55°C in KPB buffer by measuring initial reaction rates. Substrate (sucrose, 0.005–0.5 M) was incubated with 36 ng N.BfrA protein (quantified using Bradford method and ImageJ (http://imagej.nih.gov/ij/)) for 10 min and the reactions were terminated by boiling for 2 min. The amount of glucose was determined by the glucose assay kit and confirmed by HPLC analysis (Aminex HPX-42 column, 300×7.8 mm, Bio-Rad, USA). Experiments were performed in triplicate and the kinetic parameters were determined by fitting the data to the standard Michaelis–Menten equation through GraFit 7.0 (http://www.erithacus.com/grafit/).

### Protein identification by mass spectrometric peptide mapping and N-terminal amino acid sequencing

For mass spectrometry, Coomassie-stained protein bands were manually excised from gels. In-gel digestion was conducted using trypsin and chymotrypsin. Digested peptides were analyzed by Shimazu LCMS-IT-TOF mass spectrometer coupled with 2-D nano LC system (Shimadzu, Japan), mass spectral data were searched against the NCBInr database, and protein was identified using the MASCOT program (http://www.matrixscience.com/). For protein N-terminal identification, the SDS–PAGE protein band that corresponded to the active fragment obtained from native electrophoresis was transferred to a polyvinylidene fluoride membrane (Millipore, USA). The N-terminal residues of the band with sucrose hydrolytic activity were sequenced by the Edman degradation method. Protein sequence databases were searched using the BLAST software at the National Center for Biotechnology Information server (NCBI, http://www.ncbi.nlm.nih.gov).

### cDNA cloning and recombinant protein expression

The cDNA encoding BfrA (*bfrA*FS4) was cloned by reverse transcription PCR. Total RNA was isolated as previously described [23] using the TRIzol reagent (Invitrogen, USA). Primers used in cloning procedures are listed in Table 1. The first cDNA strand was synthesized using the Revert Aid H Minus First Strand cDNA Synthesis Kit (Fermentas, USA) and an oligo (dT) primer. The cDNA fragments of BfrAFS4 were amplified by PCR using N-TER and G1 R primers. N-TER was designed based on the N-terminal sequencing result of the purified N.BfrA, and G1 R was designed based on the conserved sequences of *A. oryzae* fructosidases from GenBank. The 3′- and 5′-ends of the cDNA were amplified using the 3′/5′-Full RACE Core Set by following the manufacturer's protocols (Invitrogen, USA). For the cDNA 3′-end amplification, two sets of primers were used; one set consisted of an oligo (dT)-3′adaptor primer and a gene-specific primer F1, and the other set was an adaptor primer (AUAP) and a gene-specific primer F2. The two sets of primers for the 5′-end amplification were R1 and 5AP and nested PCR primers R2 and AUAP. The resulting PCR products were cloned into the pMD18-T vector and sequenced. Sequence analysis and multiple alignments were performed using the BLAST Tool (http://www.ncbi.nlm.nih.gov/BLAST/).

**Table 1:** Primers used for cDNA cloning and expression vector construction.

Primers	DNA sequence	Restriction Enzyme site
Oligo (dT)	TTTTTTTTTTTTTTTTTT	
N-TER	TCCGCCATCGATTACAACGCAGCTC	
G1 R	AGTACTCARSGCAAARCGWCCATTG	
3AP	GGCCACGCGTCGACTAGTACTTTTTTTTTTTTTTTTT	
AUAP	GGCCACGCGTCGACTAGTAC	
F1	TGCCCGTAATTCTGATCTCCA	
F2	TCAATCATCGTCGACCGCA	
5 AP	GGCCACGCGTCGACTAGTACCCCCCCCCCCCCCCCC	
R1	TTCGTGGACACCACCAGAGAT	
R2	TTTCGGCTCCTGGGTTGTA	
bfr-b-S-F	CAGG|AATTCGATGAGGCTCTCAACCGCGAGTG	*Eco*R I
bfr-R1	GTCA|AGCTTGACACGCTCAGGCCAGGCTTCA	*Hind* III
bfr-p-S-F	CAGG|AATTCATGAGGCTCTCAACCGCGAG	*Eco*R I
bfr-R2	TAGCATGC|GGCCGCTTAGACACGCTCAGGCCAGG	*Not* I

The cDNA that encoded the mature fructosidase (m*bfrA*FS4) was used as a template for PCR amplification using primers bfr-b-S-F and bfr-R1 (Table 1) with *EcoR* I and *Hind* III recognition sites, and primers bfr-p-S-F and bfr-R2 (Table 1) with *EcoR* I and *Not* I recognition sites. The amplification fragments were sequenced and cloned into the corresponding sites of pET26b and pPIC9K expression vectors.

The pET26/m*bfrA*FS4 was overexpressed in *E. coli* BL21 by isopropyl-β-D-thiogalactopyranoside induction at 16°C for 20 h. Cells were harvested and lysed. The overexpressed recombinant fructosidase was purified by Ni^2+^-NTA affinity chromatography.

The *P. pastoris* expression plasmids (pPIC9K/m*bfrA*FS4) were linearized by *Stu I* and transformed into *P. pastoris* KM71 cells by electroporation based on the protocols in the *Pichia* Expression Kit (Invitrogen, USA). The transformants were cultivated at 30°C in the BMGY medium. When cell density reached 2.0 OD to 6.0 OD at 600 nm, the cells were harvested and resuspended in the BMMY medium using 1/5 to 1/10 of the original culture volume. Methanol was added to a final concentration of 0.5% every 24 h to maintain induction for 48 h. The supernatant was then collected as the crude enzyme solution and applied to ammonium sulfate precipitation (85% saturation) for one-step purification.

### pH, temperature effect, and substrate specificity

Optimum temperature was determined by assaying the β-fructosidase activity from 35°C to 75°C in KPB at a constant pH (pH 7.0), and the optimum pH was determined at a constant temperature (55°C) under different pH conditions (pH 3.0 to 11.0). The pH and thermal stability was determined by assaying the residual β-fructosidase activity at standard reaction condition after incubating the native and recombinant enzymes for 24 h at various pH values from 3.0 to 11.0 and at temperatures that ranged from 35°C to 75°C for 2 h. The hydrolytic substrate specificity of native and recombinant enzymes was tested at optimum reaction conditions on 50 mM of oligosaccharides [sucrose (Glc α-1, 2-β Fru), raffinose (Gal α-1, 6- Glc α-1, 2-β Fru), turanose (Glc α-1, 3-α Fru), leucrose (Glc α-1, 5-β Fru), and palatinose (Glc α-1, 6-β Fru)], and product formation was analyzed by thin-layer chromatography (TLC) using butanol–enthanol-distilled water (5∶3∶2 [v/v/v]) as a mobile phase. The kinetic parameters for turanose (0.005–1 M) and leucrose (0.01–1 M) were determined as described above. For the reaction with raffinose (0.005–0.25 M) as the substrate, the release of melibiose instead of glucose was measured through HPLC analysis.

### Isolation and identification of FOS

The transfructosylation reaction was performed at 50°C for 5 h in a mixture that contained 5 µL of enzyme and 33% of sucrose in 50 mM KPB (pH 7.0). The products were analyzed by TLC using butanol–enthanol-distilled water (5∶3∶2 [v/v/v]) as a mobile phase and detected by spraying with 0.05% (w/v) 3,5-dihydroxytoluene in 20% (v/v) sulfuric acid and heating at 120°C. The yields were quantified using ImageJ. For further structural analysis, oligosaccharides were separated using the Bio-Gel P2 column (1.6×100 cm). The products were eluted with distilled water at a flow rate of 125 µL/min, and the fractions were collected and analyzed by TLC and MS spectrum. The products that could not be separated by the Bio-Gel P2 column were purified by TLC recovery. Mass spectra were recorded on a LCMS-IT-TOF instrument (Shimadzu, Japan) equipped with an ESI source in positive ion mode at a resolution of 10,000 full width at half-maximum. The chemical structures of oligosaccharides were determined by nuclear magnetic resonance (NMR) spectroscopy. The NMR data were collected at 26°C in D_2_O on a Bruker DRX Advance-600 spectrometer (Bruker Biospin AG, Fallanden, Switzerland), including ^1^H, ^13^C, correlation spectroscopy, total correlation spectroscopy, heteronuclear single-quantum coherence, and heteronuclear multiple-bond correlation spectra.

### N-glycosylation site prediction and analysis

The N-glycosylation sites of BfrA were predicted by NetNGlyc 1.0 (http://www.cbs.dtu.dk/services/NetNGlyc/). To analyze the N-glycosylation condition, the purified N.BfrA and *Pichia*-expressed BfrA (P.BfrA) were denatured and incubated with PNGase F overnight at 37°C, and the deglycosylated samples were analyzed on SDS gel with *E. coli*-expressed recombinant BfrA (E.BfrA) as a control. The N-glycosylation sites of the N.BfrA and P.BfrA were analyzed by LC–MS/MS. N-glycans were released by PNGase F after in-gel chymotrypsin digestion of the Coomassie-stained protein samples. The resulting deglycosylated peptides were desalted, concentrated by the zip-tip column, and analyzed using an LTQ Orbitrap mass spectrometer (Thermo Scientific, Bremen, Germany) operated through the Xcalibur software (Thermo Fisher Scientific, USA) coupled with nanospray LC (Eksigent Technologies Inc., CA). The N-glycosylation site information was derived from the MS/MS spectra by searching online against the NCBInr database.

To analyze the role of N-glycosylation structure in the function of the BfrA, a tertiary structure was generated using homology modeling based on a crystal structure of the *Aspergillus japonicas* fructosyltransferase (pdb3LF7A, 62% sequence identity) by the SwissModel Automatic Modelling server (http://swissmodel.expasy.org/workspace/index.php?func=modelling_simple1&userid=USERID&token=TOKEN) and displayed by Pymol software (http://www.pymol.org/).

## Results and Discussions

### N.BfrA purification and N-terminal identification

**Figure 1:**
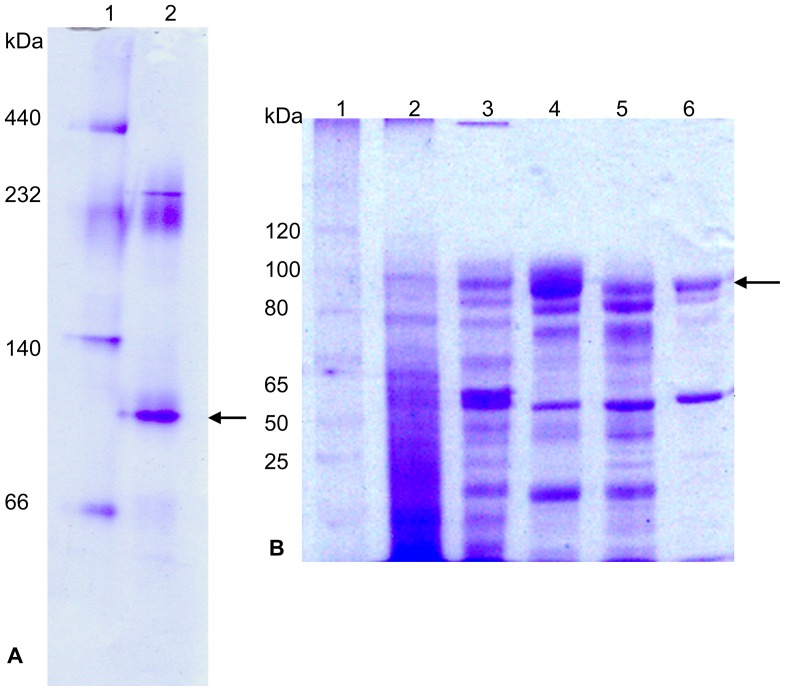
Separation of BfrA from *A. oryzae* SF4. (A) Native gradient PAGE analysis of purified N.BfrA. Lane 1, molecular mass standards; lane 2, partially purified fructosidase was subjected to electrophoresis on 5% to 15% native gradient polyacrylamide gels. The targeted protein band with fructosidase activity was indicated by an arrow. (B) SDS–PAGE for all steps of purification of BfrA from *A. oryzae* SF4. Lane 1, molecular mass standards; lane 2, crude enzyme; lane 3, protein fraction after dialysis; lane 4, protein fraction from DEAE–Sepharose fast flow step; lane 5, protein fraction from Superdex 200 step; lane 6, protein fraction from Native–PAGE purification. Protein bands were visualized by staining with Coomassie blue. Target protein band was indicated by an arrow.

*A. oryzae* has been widely used in the food industry for production of FOS. However, the *A. oryzae* fructosidases have been mainly reported at the gene level. Thus, further characterization of these enzymes is necessary. In this study, an *A. oryzae* fructosidase was purified from the crude extract of *A. oryzae* FS4 culture, and the results of each purification step are reported in Table 2. The molecular mass of fructosidase was estimated to be approximately 100 kDa (Fig. 1A) using native PAGE, and 110 kDa by gel filtration (S1 Figure). They are very close to that estimated by SDS–PAGE (95 kDa; Fig. 1B), which suggests that the native form of the enzymes is a monomer. The purified fraction exhibits two bands, namely, one band with an apparent molecular mass of approximately 95 kDa and the other band with an apparent molecular mass of 40 kDa (Fig. 1B, lane 6). The 21 peptides of the purified N.BfrA protein (95 kDa form, indicated by an arrow in Fig. 1B) were identified by MS, and the protein fingerprint mapping matched the deposited fructosyltransferase protein (UnitProtKB accession No. Q27J21). Meanwhile, the contaminant protein was identified as an unknown protein. The N-terminal sequence of the purified enzyme was estimated as (A/S/A) IDYNAAPPNL with microheterogeneity at the initial amino acids, suggesting various cleavages of a signal peptide at the initial site [24].

**Table 2:** Purification procedures of β-fructosidase from *A. oryzae* SF4.

Steps	Volume	Activity	Total	Protein	Specific	Yield	Purification
	(ml)	(U/ml)	activity	concentration	activity	(%)	fold
			(Units)	(mg/ml)	(U/mg)		
Crude enzyme	175	37.9	6640.5	5.5	6.9	100	1
(NH_4_)_2_SO_4_ 50–85%	30	135.1	4054.1	8.2	16.4	61.1	2.4
DEAE-Fast Flow/ Superdex-200	25	119.1	2977.5	3.1	38.4	44.8	5.6
Native-Page recovery	1	1022.0	1022.0	0.55	1858.2	15.4	269.3

### BfrA cloning and expression

The BfrA coding sequence with a size of 1860 base pairs was amplified from *A. oryzae* FS4 and sequenced (GenBank accession No. KF765438). Based on software prediction (http://www.cbs.dtu.dk/services/SignalP/), the BfrA full-length protein contained a signal peptide (MRLSTASALVTSQAAYAASA), which consisted of the N-terminal sequence result, and shared 88% identity with those of reported extracellular invertases. The full-length protein with a predicted pI value of 4.71 exhibited a calculated molecular mass of 67216 Da (molecular mass of mature protein is 65298). The amino sequences deduced from the nucleotide sequence were 90% similar to the extracellular invertase from *A. oryzae* RIB40 and *A. oryzae* 3.042, GH32 superfamily member from *Aspergillus flavus* NRRL3357, and fructosyltransferase from *A. oryzae* (GenBank Accession Nos. XP_003190558.1, EIT76425.1, XP_002383662.1, and ABW87267.1) (S2 Figure).

**Figure 2:**
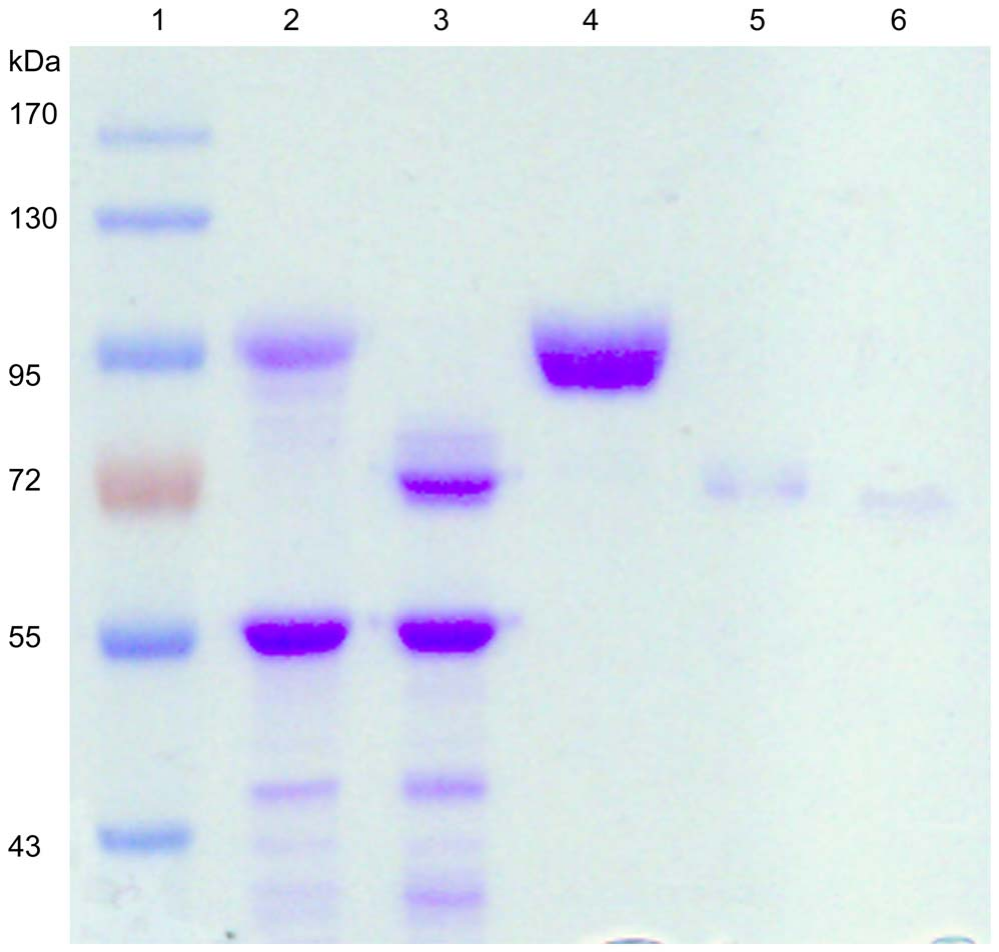
Glycosylation analysis of native and recombinant BfrAs. Lane 1, molecular mass standards; lane 2, N.BfrA purified from *A. oryzae* SF4; lane 3, PNGase F treatment of N.BfrA; lane 4, purified P.BfrA; lane 5, PNGase F treatment of P.BfrA; lane 6, purified E.BfrA.

Considering the multiple purification steps for N.BfrA isolation from *A. oryzae*, and to avoid simultaneous separation with other contamination proteins, the enzyme was overexpressed in *E. coli* and *P. pastoris*, and its biochemical properties were subsequently determined in detail. Recombinant BfrAs were purified to homogeneity, and the purity was confirmed by the SDS–PAGE gel (Fig. 2). The SDS–PAGE result indicates approximate relative masses of 95 and 70 kDa for the P.BfrA and E.BfrA, respectively (Fig. 2, lanes 4 and 6). The purified N.BfrA also showed a molecular mass of approximately 95 kDa (Fig. 2, lane 2), which is approximately 30 kDa higher than the predicted mature protein molecular weight (approximately 65 kDa). Using software (http://web.expasy.org/cgi-bin/glycomod), 13 potential N-glycosylation sites (Asn-X-Ser/Thr) were identified, and the glycosylation condition of the purified enzymes was confirmed by the PNGase F treatment (Fig. 2, lanes 2 and 3). After incubation with PNGase F, the deglycosylated N.BfrA (Fig. 2, lane 3) and P.BfrA (Fig. 2, lane 5) showed mobility similar to that of E.BfrA (Fig. 2, lane 6), which suggests that both N.BfrA and P.BfrA are N-glycosylated. The total percentage of carbohydrate was determined as approximately 31% of the total mass of the protein.

### Influence of temperature and pH

**Figure 3:**
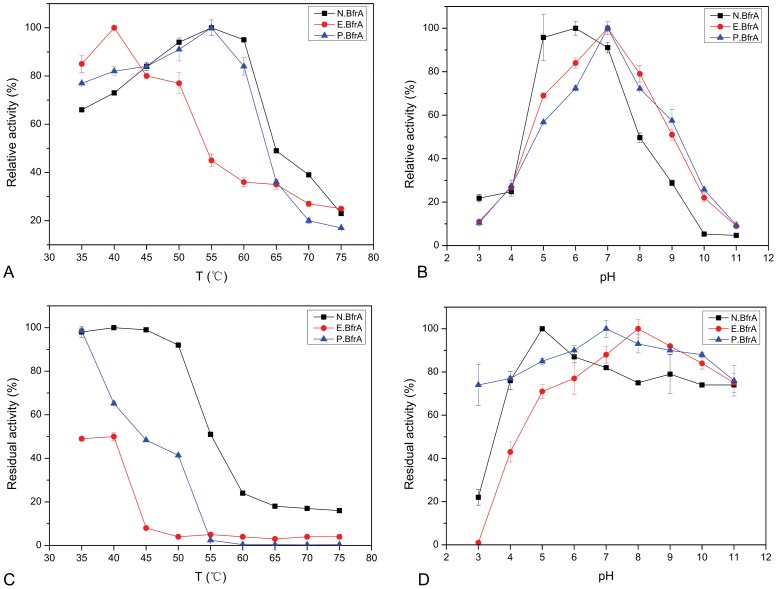
Effects of temperature and pH on fructosidase activity. (A) Temperature- and. (B) pH-dependency activity curves. (C) Thermostability curve, residual activity after 2 h incubation at different temperatures from 35°C to 75°C. (D) pH-stability curve, residual activity after 24 h incubation in different pH buffers from pH 3 to 11 at 4°C. N.BfrA (square), P.BfrA (circle), and E.BfrA (triangle) were analyzed simultaneously, and each data point represents the mean ± SD of one individual experiment performed in triplicate.

To compare the native and recombinant BfrAs, thermo- and pH-dependent activities, as well as thermo and pH stabilites, were analyzed. The purified native and recombinant BfrAs showed very high activities of 3717 (N.BfrA), 3637 (P.BfrA), and 682 U/mg (E.BfrA) using sucrose as substrate. The optimum activity of N.BfrA and P.BfrA was obtained at 55°C; they retained ca. 80% of the activity up to 60°C (Fig. 3A). However, E.BfrA had an optimum temperature as low as 40°C. At 60°C, E.BfrA only kept less than 40% activity, which was much lower than those of N.BfrA and P.BfrA. The effect of pH on fructosidase activity was tested with buffers of pH values that ranged from 3 to 11. The native enzyme was highly active at pH 5.0 to 7.0 with an optimum pH of 6.0 (Fig. 3B), and it gradually became inactivated at pH below 5.0 and lost almost 50% of its activity at pH over than 8.0, These results are consistent with those of extracellular invertase from *Saccharomyces cerevisiae* [25]. The pH-dependent activation profiles of the two recombinant BfrAs were almost indistinguishable, and their optimum pH values were both at pH 7.0, which was higher than that of N.BfrA. Using the heterologous expression of BfrA, the optimal pH of the enzyme was increased from pH of 6.0 to 7.0, which enhanced the suitability of the enzyme for the food industry [26].

For thermostability profiles, Fig. 3C shows distinct differences in enzyme residual activity. N.BfrA was stable under 50°C for 2 h, and temperatures greater than 55°C inactivated the enzyme. P.BfrA was stable at 35°C for 2 h, and its residual activity still remained at 45% even after 2 h of incubation under 50°C. However, E.BfrA was not as thermostable as the other two enzymes. It was stable within a range from 0°C to 4°C (data not shown), only retained 50% activity at 35°C after 2 h of incubation, and completely lost its activity after 2 h of incubation at 50°C. For pH stability profiles, N.BfrA retained ca. 80% of its initial activity after 24 h of incubation at moderately acid, neutral, and basic pH values (from 4.0 to 11.0) at 4°C (Fig. 3D). However, it retained only 20% of the initial activity at pH 3.0 (Fig. 3D). P.BfrA exhibited a broader pH stability and retained >80% of its initial activity with a pH range of 3.0 to 11.0. E.BfrA exhibited much lower stability in moderately acidic buffer (pH 4.0 to 6.0) and showed no detectable activity at pH 3.0. Nevertheless, both N.BfrA and P.BfrA retained most activity at pH 4.0 to 11.0, especially after incubation with basic buffers. This result suggested that the enzymes show tolerance within a wide pH range.

### Kinetic properties and substrate specificity

The *Km* and *k_cat_* values for total enzyme activities were summarized in Table 3. Michaelis–Menten-type kinetics was observed with different substrates (sucrose, turanose, leucrose and raffinose) with minor sucrose and substrate inhibition. The Michaelis constant *Km* (37.19±5.28 mM) for sucrose was close to that (38 mM) of invertase from *S*. *cerevisiae* [27], slightly higher than that (11 mM) of β-fructofuranosidase from *Bifidobacterium adolescentis* G1 [28], but much lower than that (0.227 M and 0.06 M) of invertase from *Rhodotorula glutinis* [29], and from *A. niger* [30], respectively. The molecular activity *k_cat_*, (1.0016±0.039×10^4^ s^−1^) and catalytic coefficiency *k_cat_*/*Km* (269.34±39.68 mM^−1^ s^−1^), however, were much higher than those (*k_cat_* and *k_cat_*/*Km*, 59.1 and 5.37 mM^−1^ s^−1^) of β-fructofuranosidase from *B. adolescentis* G1 [27]. These results suggested that BfrA had high catalytic activity and could be potentially used in industrial production.

**Table 3 a:** Kinetic parameters of N.BfrA with different substrates.

Substrates	Vmax	*Km* (mM)	*k_cat_* b(s^−1^)	*k_cat_*/*Km* (mM^−1^ s^−1^)
	(mM/µg of protein/min)			
Sucrose	127.50±4.87	37.19±5.28	1.0016±0.039×10^4^	269.34±39.68
Turanose	56.97±2.68	741.89±64.92	4.51±0.21×10^3^	6.08±0.60
Leucrose	3.06±0.48	216.85±28.06	2.53±0.13×10^2^	1.17±0.16
Raffinose	126.27±8.68	108.20±12.20	1.044±0.072×10^4^	96.48±13.30

a The results are presented as the means ± SD.

b A molecular mass of 95 kDa were used for the N.BfA.

**Figure 4:**
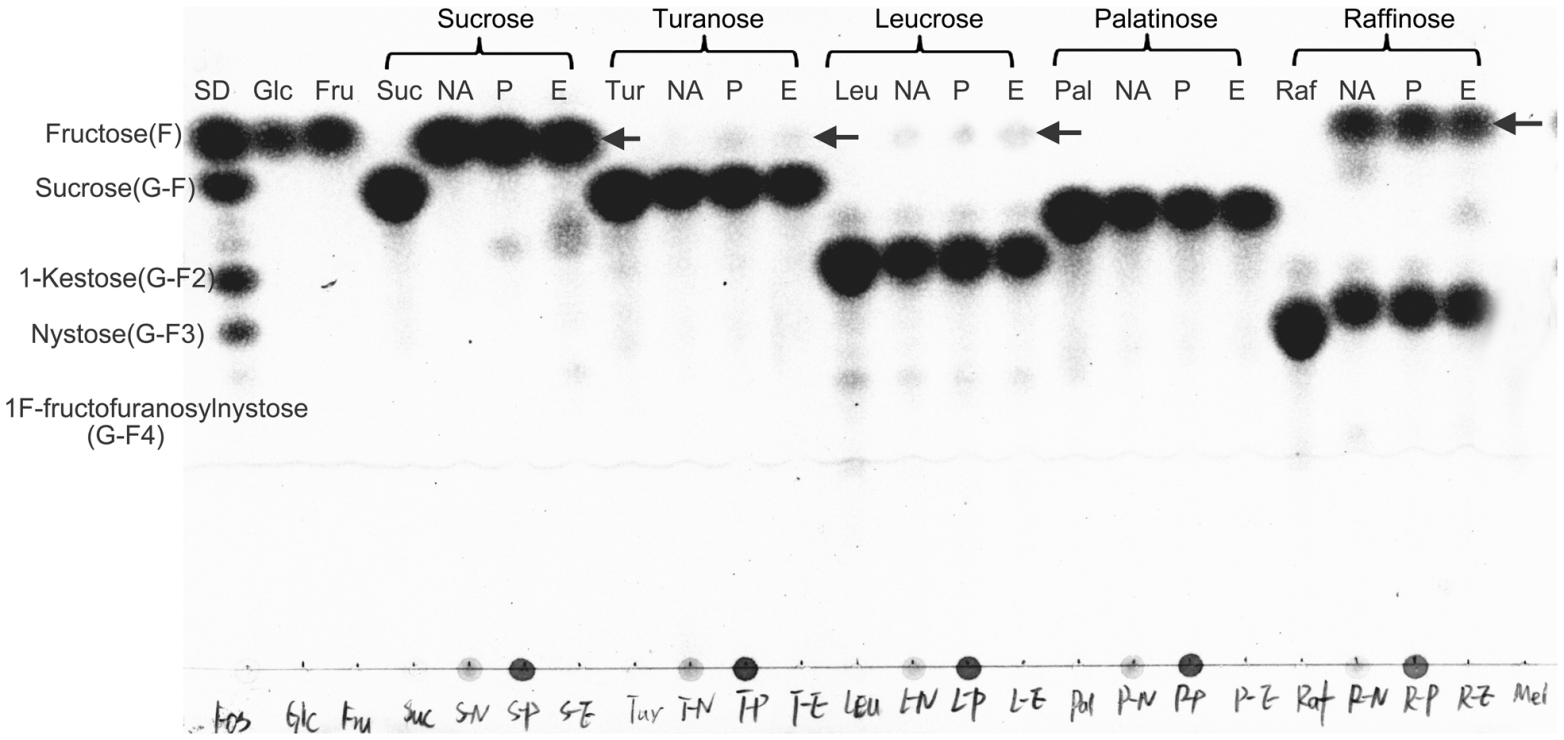
Hydrolytic substrate specificity of native and recombinant BfrAs. Each substrate [50 µmol; sucrose (Suc), raffinose (Raf), turanose (Tur), leucrose (Leu), and palatinose (Pal)] were detected with equal amount of N.BfrA, P.BfrA, and E.BfrA at optimal reaction condition. The hydrolytic products were indicated by arrows. SD, Standards of FOS; Glc, glucose; Fru, fructose; NA, hydrolytic reaction catalyzed by N.BfrA; P, hydrolytic reaction catalyzed by P.BfrA; E, hydrolytic reaction catalyzed by E.BfrA.

Substrate specificity analysis for both native and recombinant enzymes was conducted under optimum reaction conditions. The enzymes were tested with a number of substrates, which included sucrose, raffinose, turanose, leucrose, and palatinose (Fig. 4). The release of fructose in each of the substrates was confirmed by TLC (Fig. 4, indicated by arrows) and mass spectrometry analysis (S3 Figure). The BfrAs showed high activity against sucrose and raffinose (Glc α-1, 2-α Fru, and Gal α-1, 6- Glc α-1, 2-β Fru), contrary to its lower activity against turanose (Glc α-1, 3-α Fru) and leucrose (Glc α-1, 5-β Fru). No activity could be detected with palatinose (Glc α-1, 6-β Fru) as substrate. Most of the reported fructosidases from a variety of microorganisms could not hydrolyze other fructosyl bonds than (1-2)-linked fructose. By contrast, the *Arxula adeninivorans* show low hydrolytic activity for (1-3)-linked turanose [31] and a β-fructofuranosidase from *Xanthophyllomyces dendrorhous* can hydrolyze β(1-6)-linked palatinose [21]. However, to the best of the authors' knowledge, such broad substrate specificity [degradation of (1-2)-, (1-3)-, and (1-5)-linked FOS] of fructosidase has not been described.

Michaelis constants (*Km*), molecular activity (*k_cat_*), and catalytic coefficiency (*k_cat_*/*Km*) for each substrate were calculated (Table 3). In all substrates reactions, the *Km* value of sucrose is the lowest, and Vmax and *k_cat_*/*Km* of sucrose were the highest, which indicated that BfrA had the highest affinity to sucrose and hydrolyzed sucrose at the greatest rate. The *Km* value of trisaccharide raffinose was about 2 folds higher that of sucrose, but much lower than those of turanose and leucrose. The Vmax and *k_cat_* values of raffinose and sucrose were nearly the same. This result suggested enzyme preferred substrates containing 1, 2-linked fructose to those containing 1, 3-linked and 1, 5-linked fructose, which was consistent with the result from TLC analysis.

### FOS identification

**Figure 5:**
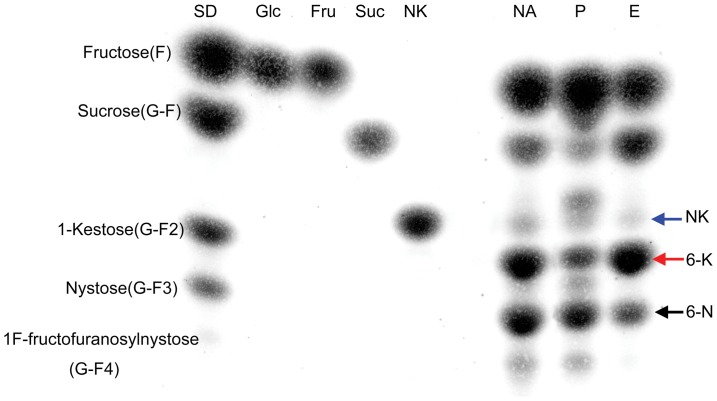
TLC analysis of FOS from transglycosylation reactions. The transglycosylation reactions were catalyzed by native and recombinant BfrAs incubated with 33% sucrose at optimal reaction condition for 5 h. The FOS were separated by Bio-Gel P2 column. SD, FOS standards; Glc, glucose; Fru, fructose; Suc, sucrose; NK, neokestose; NA, transglycosylation reaction catalyzed by N.BfrA; P, transglycosylation reaction catalyzed by P.BfrA; E, transglycosylation reaction catalyzed by E.BfrA. The neokestose (NK), 6-kestose (6-K), and 6-nystose (6-N) were indicated by blue, red, and dark arrows, respectively.

The other interesting feature of this BfrA is that it can yield levan- and neolevan-type β-(2-6)-linked FOS. At high sucrose concentration (33%), both the native and recombinant BfrAs performed transfructosylation reactions. The composition of the oligosaccharide fractions separated by the Bio-Gel P2 column and TLC recovery was identified by Mass (S5–S7 Figures) and NMR analyses (S8–S22 Figures). The product mixture consisted of approximately 56% FOS (quantified by ImageJ and confirmed by HPLC, S4 Figure) with GF2 (26.2%), GF3 (20.4%), and GF4 (9.2%). Two types of fructose-based GF2 oligosaccharides were detected and identified as levan-type 6-kestose [β-D-Fru-(2-6)-β-D-Fru-(2-1)-α-D-Glc] (Fig. 5, red arrow; S6 Figure, S13–S17 Figures) and neolevan-type neokestose [β-D-Fru-(2-6)-α-D-Glc-(1-2)-β-D-Fru] (Fig. 5, blue arrow; S5 Figure, S8–S12 Figures) by Mass spectrometry and NMR. Moreover, the 6-kestose accounted for around 19% of the total products. The GF3 was identified as levan-type 6-nystose [β-D-Fru-(2-6)-β-D-Fru-(2-6)-β-D-Fru-(2-1)-α-D-Glc] by NMR analysis (S18–S22 Figures), and the structure of GF4 was still not confirmed. The levan- (^6^F-FOS, such as 6-kestose) and neolevan-type (^6^G-FOS, such as neokestose) FOS exhibited higher prebiotic activity than the usual inulin-type FOS [3], [5]. The enzymatic synthesis of the levan-type FOS has been reported in yeast, such as *S. cerevisiae* [32], *Schwanniomyces occidentalis* [33], and *R. dairenensis* [34], and in fungi, such as *Thermoascus aurantiacus*. Neolevan-type FOS have been reported to be synthesized by a β-fructofuranosidase from *X. dendrorhous* [10], [21]. Although *Aspergillus* spp. has been considered as the main industrial producer of FOS, its fructosyltransferase was only reported to produce the inulin-type [β-(2-1)-linked] FOS [1], [9]. Additionally, for all the GH32 family enzymes reported from this organism, limited data on the synthesis of β-(2-6)-linked fructose polymers have been published. In the present study, this novel fructosidase can be potentially applied for the development of new prebiotic oligosaccharides, and further protein structural analysis is necessary to fully understand its special biological function.

### N-glycosylation site identification and distribution

As described in the previous sections, the deglycosylated value of N.BfrA and P.BfrA was approximately 70 kDa, as estimated by SDS–PAGE (Fig. 2). This value was very close to the calculated molecular mass, which suggested the presence of N-linked glycans. The N-glycosylation condition of N.BfrA and P.BfrA was analyzed by MS, and the assignment of N-linked glycopeptides was performed using chymotryptic peptides before and after PNGase F treatment. MS results confirmed that all but three (Asn^29^, Asn^35^, and Asn^250^) of the 13 putative glycosylation sites were N-glycosylated on N.BfrA. Except for the three non-glycosylated sites, the other three potential N-glycosylation sites (Asn^164^, Asn^314^, and Asn^320^) on P.BfrA were not detected for glycosylation, which may have resulted from the lower recovery rate of MS (protein sequence coverage was 74% for P.BfrA) or different glycosylation patterns between *A. oryzae* and *P. pastoris*.

Glycans, as hydrophilic polymers, usually contribute to proper folding, prevent self-aggregation, improve solubility, and increase stability against proteolysis [35]. The absence of the N-glycan on E.BfrA affected its proper folding, which resulted in the lower optimal activity (682 U/mg) than those of the glycosylated N.BfrA and P.BfrA (3717 and 3637 U/mg, respectively). Moreover, the covalent binding of glycans on the protein surface may inherently enhance the thermostability of proteins. The absence of N-linked oligosaccharides on E.BfrA resulted in a significant decrease in thermostability comparison with the glycosylated N.BfrA and P.BfrA enzymes (Fig. 3A and 3C). This result suggested that N-glycosylation is a major determinant of the enzyme to resist thermal inactivation. The function of carbohydrate on the thermostability of various proteins has been previously reported [10], [11], [12], [13]. Although protein-bound oligosaccharides generated by *P. pastoris* are high mannose-type with an average of 8 to 12 mannose residues, which might be different from the structure of the oligosaccharide chains on the native enzymes, P.BfrA exhibits optimal activity and thermostability similar to those of N.BfrA. This result is consistent with those of previous studies, in which the degree of thermal stabilization was shown to be mainly dependent on the position of the glycosylation sites, but very weakly on the size of the glycans [35]. These results indicated that the presence of oligosaccharide chains on both N.BfrA and P.BfrA is essential in maintaining the enzyme proper structure and has a remarkable stabilizing effect on enzyme under high temperature conditions.

**Figure 6:**
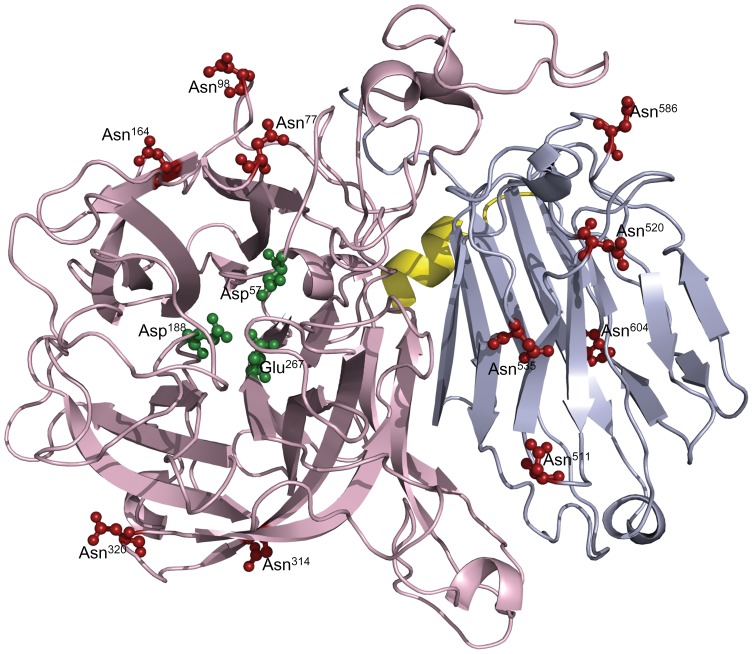
N-glycosylation sites mapping on *A. oryzae* FS4 BfrA tertiary structure model. The tertiary structure model was generated using the crystal structure of *A. Japonicus* fructosyltransferase (pdb3LF7A) as a template. Active site residues and confirmed glycosylation sites are shown in green and red ball-and-stick representation, respectively. The N-terminal β-propeller (residues 21–435), a C-terminal β-sandwich (446–618) domain, and a 9-residue short α-helix (436–445) linkage are shown in pink, gray, and yellow, respectively. The model was constructed by SwissModel Automatic Modelling server, and the image was generated by PyMOL program.

A fructosidase three-dimensional model was generated by homology modeling (SwissModel Automatic Modelling server) using the published crystallographic structure of the *A. Japonicus* fructosyltransferase (pdb3LF7A) as a template. Similar to the published structure [36], the modeling 3-D structure of BfrA comprised two domains, namely, the catalytic domain that contained a five-blade propeller and the β-sandwich domain. MS confirmed the presence of 10 N-glycosylation sites, eight of which occurred on the random coil (Asn^77^, Asn^98^, Asn^320^, Asn^511^, Asn^520^, Asn^535^, Asn^586^, and Asn^604^) (Figure. 6) at both β-propeller and β-sandwich domains. The other two sites (Asn^164^ and Asn^314^) were located on two different β-sheets at the β-propeller domain. All ten glycosylation sites were exposed to the surface of the protein structure and distant from the catalytic site (formed by Asp^57^, Asp^188^, and Glu^267^) in the central cavity. This prediction agrees with the fructosidase biochemical properties, because it showed that the carbohydrate in the structure did not affect the substrate specificity, but the optimal activity and thermostability of the enzymes.

## Supporting Information

S1 Figure **Determination of molecular weight of N.BfrA by Sephadex G-200 gel filtration.** Sephadex G-200 (1.5×87 cm) column was equilibrated and eluted with 0.05 M Tris, 0.15 M NaCl buffer (pH 7.2). Calibration curve was constructed by plotting the K_av_ value for each protein standard in Gel filtration calibration kits (GE Healthcare, USA) against its molecular weight on the logarithmic scale. Standard proteins were: (1) Ovalbumin (44, 000), (2) Conalbumin (75, 000), (3) Aldolase (158, 000), (4) Ferritin (440, 00), (5) Thyroglobulin (669, 000). The red square indicates the N.BfrA.
(DOCX)

S2 Figure **Comparison of the amino acid sequence of BfrA with those of other fructosyltransferase and invertases from** ***Aspergillus spp.*** EIT76425.1 (GenBank): extracellular invertase from *A. oryzae* 3.042; XP_002383662.1 (NCBI Reference Sequence): Glycosyl hydrolases family 32 superfamily from *A. flavus* NRRL3357; XP_003190558.1 (NCBI Reference Sequence): extracellular invertase from *A. oryzae* RIB40; ABW87267.1 (GenBank): fructosyltransferase from *A. oryzae*. Asterisks indicate identical residues.
(DOCX)

S3 Figure **Hydrolytic substrate specificity analysis.** A: The chromatogram of the reaction products by HPLC. The reactions were performed used N.BfrA (36 ng) incubated with 1 M substrates [sucrose (Suc), raffinose (Raf), turanose (Tur), and leucrose(Leu)] for 10 min at 55°C. The reactions were analyzed by HPLC through an Aminex HPX-42 column (300×7.8 mm, Bio-Rad, USA). The peaks corresponding to sucrose (Suc), glucose (Glc), fructose (Frc), raffinose (Raf), turanose (Tur), leucrose (Leu), and FOS were indicated. B: Mass spectra of hydrolysis products (fructose) of reactions. The fractions a), b), c), and d) in A were collected and analyzed by LC/MS-IT-TOF (Shimadzu, Japan) in positive ion mode.
(DOCX)

S4 Figure **The time course of FOS production with N.BfrA from** ***A. oryzae*** **using 1M sucrose concentration.** The amount of FOS was analyzed by HPLC through an Aminex HPX-42 column (300×7.8 mm, Bio-Rad, USA). Experiments were performed in triplicate, and data points represent the mean ± SD.
(DOCX)

S5 Figure **Mass data of compound identified as neokestose.**
(DOC)

S6 Figure **Mass data of compound identified as 6-kestose.**
(DOCX)

S7 Figure **Mass data of compound identified as and 6-nystose.**
(DOCX)

S8 Figure **^1^H NMR spectrum of neokestose.**
(TIF)

S9 Figure **^13^C NMR spectrum of neokestose.**
(TIF)

S10 Figure **COSY spectrum of neokestose.**
(TIF)

S11 Figure **HMBC spectrum of neokestose.**
(TIF)

S12 Figure **HSQC spectrum of neokestose.**
(TIF)

S13 Figure **^1^H NMR spectrum of 6-kesose**
(TIF)

S14 Figure **^13^C NMR spectrum of 6-kesose.**
(TIF)

S15 Figure **COSY spectrum of 6-kesose.**
(TIF)

S16 Figure **HMBC spectrum of 6-kesose.**
(TIF)

S17 Figure **HSQC spectrum of 6-kesose.**
(TIF)

S18 Figure **^1^H NMR spectrum of 6-nystose.**
(TIF)

S19 Figure **^13^C NMR spectrum of 6-nystose.**
(TIF)

S20 Figure **COSY spectrum of 6-nystose.**
(TIF)

S21 Figure **HMBC spectrum of 6-nystose.**
(TIF)

S22 Figure **HSQC spectrum of 6-nystose.**
(TIF)
