# Supplementary material for: Purification, Cloning, Characterization, and N-Glycosylation Analysis of a Novel β-Fructosidase from Aspergillus oryzae FS4 Synthesizing Levan- and Neolevan-Type Fructooligosaccharides
Source: PLoS One. 2014 Dec 12;9(12):e114793. doi: 10.1371/journal.pone.0114793 (PMC4264766; doi:10.1371/journal.pone.0114793)
Supplement: S2 Figure — Comparison of the amino acid sequence of BfrA with those of other fructosyltransferase and invertases from Aspergillus spp. EIT76425.1 (GenBank): extracellular invertase from A. oryzae 3.042; XP_002383662.1 (NCBI Reference Sequence): Glycosyl hydrolases family 32 superfamily from A. flavus NRRL3357; XP_003190558.1 (NCBI Reference Sequence): extracellular invertase from A. oryzae RIB40; ABW87267.1 (GenBank): fructosyltransferase from A. oryzae. Asterisks indicate identical residues. (DOCX) [file pone.0114793.s002.docx]

EIT76425.1 1 MKLSTASALVTSQAAYAASAIDYNAAPPNLSTLANGSLYDTWRPRAHILPPNGRIGDPCG
XP_002383662.1 1 MKLSTASALVTSQAAYAASAIDYNAAPPNLSTLANGSLYDTWRPRAHILPPNGRIGDPCG
BfrA 1 MRLSTASALVTSQAAYAASAIDYNAAPPNLSTLANGSLYDTWRPRAHILPPNGRIGDPCG
XP_003190558.1 1 MKLSTASALVTSQAAYAASAIDYNAAPPNLSTLANGSLYDTWRPRAHILPPNGRIGDPCG
ABW87267.1 1 MKLSTASALVTSQAAYAASAIDYNAAPPNLSTLANGSLYDTWRPRAHILPPNGRIGDPCG
consensus 1 *.**********************************************************


EIT76425.1 61 HYTDPDTGLFHVGFLYNGSGIAGATTDDMVRFRDLNPNGSQFITPGGKNDPVAVFDGSVI
XP_002383662.1 61 HYTDPDTGLFHVGFLYNGSGIAGATTDDMVRFRDLNPNGSQFITPGGKNDPVAVFDGSVI
BfrA 61 HYTDPDTGLFHVGFLYNGSGIAGATTDDMVRFRDLNPNGSQFIMPGGKNDPVAVFDGSVI
XP_003190558.1 61 HYTDPDTGLFHVGFLYNGSGIAGATTDDMVRFRDLNPNGSQFIMPGGKNDPVAVFDGSVI
ABW87267.1 61 HYTDPDTGLFHVGFLYNGSGIAGATTDDMVRFRDLNPNGSQFIMPGGKNDPVAVFDGSVI
consensus 61 *******************************************.****************


EIT76425.1 121 PKGIDGKPTLLYTSVTSLPIHWSIPYNPGAETQSLAVTSNGGRNFTKLDRPPVIPLPPSD
XP_002383662.1 121 PKGIDGKPTLLYTSVTSLPIHWSIPYNPGAETQSLAVTSNGGRNFTKLDRPPVIPLPPSD
BfrA 121 PKGIDDKPTLLYTSVTSLPIHWSIPYNPGAETQSLAVTSNGGRNFTKLDRPPVIPLPPSD
XP_003190558.1 121 PKGIDGKPTLLYTSVTSLPIHWSIPYNPGAETQSLAVTSNGGRNFTKLDRPPVIPLPPSD
ABW87267.1 121 PKGIDGKPTLLYTSVTSLPIHWSIPYNPGAETQSLAVTSNGGRNFTKLDRPPVIPLPPSD
consensus 121 *****.******************************************************


EIT76425.1 181 SDVTAFRDPYAFQSPELDAAADSAPGTWYTAISGGVHEDGPGQFLYRQDQKEMSLESWEY
XP_002383662.1 181 SDVTAFRDPYAFQSPELDAAADSAPGTWYTAISGGVHEDGPGQFLYRQDQKEMSLESWEY
BfrA 181 SDVTAFRDPYAFQSPELDAAADSAPGTWYTAISGGVHEDGPGQFLYRQDQKEMSLESWEY
XP_003190558.1 181 SDVTAFRDPYAFQSPELDAAADSAPGTWYTAISGGVHEDGPGQFLYRQDQKEMSLESWEY
ABW87267.1 181 SDVTAFRDPYAFQSPELDAAADSAPGTWYTAISGGVHEDGPGQFLYRQDQKEMSLESWEY
consensus 181 ************************************************************


EIT76425.1 241 LGLWWQEKVNTTWGNGDWAGGWGFNFETGNVFGLNEEGYSVDGEMFMTLGTEGSGTPIVS
XP_002383662.1 241 LGLWWQEKVNTTWGNGDWAGGWGFNFETGNVFGLNEEGYSVDGEMFMTLGTEGSGTPIVS
BfrA 241 LGLWWQEKVNTTWGNGDWAGGWGFNFETGNVFGLNEEGYSVDGEMFMTLGTEGSGTPIVS
XP_003190558.1 241 LGLWWQEKVNTTWGNGDWAGGWGFNFETGNVFGLNEEGYSVDGEMFMTLGTEGSGTPIVS
ABW87267.1 241 LGLWWQEKVNTTWGNGDWAGGWGFNFETGNVFGLNEEGYSVDGEMFMTLGTEGSGTPIVS
consensus 241 ************************************************************


EIT76425.1 301 QVSSIHDMLWAAGNVSNNGNVTFTPTMAGVFDWGASGYAAAGHILPATSQVSTKSGAPDR
XP_002383662.1 301 QVSSIHDMLWAAGNVSNNGNVTFTPTMAGVFDWGASGYAAAGHILPATSQVSTKSGAPDR
BfrA 301 QVSSIHDMLWAAGNVSNNGNVTFTPTMAGVFDWGASGYAAAGHILPATSQVSTKSGAPDR
XP_003190558.1 301 QVSSIHDMLWAAGNVSNNGNVTFTPTMAGVFDWGASGYAAAGHILPATSQVSTKSGAPDR
ABW87267.1 301 QVSSIHDMLWAAGNVSNNGNVTFTPTMAGVFDWGASGYAAAGHILPATSQVSTKSDAPDR
consensus 301 *******************************************************.****


EIT76425.1 361 FISFVWLTGDLFEQAKGYPTSQQNWVGTLLLPRELHIKTISNVVDNELAREEGSSWRVER
XP_002383662.1 361 FISFVWLTGDLFEQAKGYPTSQQNWVGTLLLPRELHIKTISNVVDNELAREEGSSWRVER
BfrA 361 FISFVWLTGDLFEQAKGYPTSQQNWVGTLLLPRELHIKTISNVVDNELAREEGSSWRVER
XP_003190558.1 361 FISFVWLTGDLFEQAKGYPTSQQNWVGTLLLPRELHIKTISNVVDNELAREEGSSWRVER
ABW87267.1 361 FISFVWLTGDLFEQAKGYPTSQQNWVGTLLLPRELHIKTISNVVDNELAREEESSWRVER
consensus 361 ****************************************************.*******


EIT76425.1 421 GQSGIELKTLGIDIARETREALMSGPKITEPERTSKEAGLVPFQVSPTTKFHVLTAQLSF
XP_002383662.1 421 GQSGIELKTLGIDIARETREALMSGPKITEPERTSKEAGLVPFQVSPTTKFHVLTAQLSF
BfrA 421 GQSGIELKTLGIDIARETREALMSGPKITEPERTSKEAGLVPFQVSPTTKFHVLTAQLSF
XP_003190558.1 421 GQSGIELKTLGIDIARETREALMSGPKITEPERTSKEAGLVPFQVSPTTKFHVLTAQLSF
ABW87267.1 421 GQSGIELKTLGIDIARETREALMSGPKITEPERTSKEAGLVPFQVSPTTKFHVLTAQLSF
consensus 421 ************************************************************

EIT76425.1 481 PRSARNSDLQAGFQVLSSDLESTTIYYQFSNESIIVDRSNTSAAAKTTNGIVSTNESGRL
XP_002383662.1 481 PRSARNSDLQAGFQVLSSDLESTTIYYQFSNESIIVDRSNTSAAAKTTNGIVSTNESGRL
BfrA 481 PRSARNSDLQAGFQVLSSDLESTTIYYQFSNESIIVDRSNTSAAAKTTNGIVSTNESGRL
XP_003190558.1 481 PRSARNSDLQAGFQVLSSDLESTTIYYQFSNESIIVDRSNTSAAAKTTNGIVSTNESGRL
ABW87267.1 481 PRSARNSDLQAGFQVLSSDLESTTIYYQFSNESIIVDRSNTSAAAKTTNGIVSTNESGRL
consensus 481 ************************************************************


EIT76425.1 541 RLFDLQGDVQEIETLDLTVVVDNSVLEIYANGRFALSTWARSWYKNSTDIKFYHNGAGEV
XP_002383662.1 541 RLFDLQGDAQEIETLDLTVVVDNSVLEIYANGRFALSTWARSWYKNSTDIKFYHNGAGEV
BfrA 541 RLFDLQGDAQEIETLDLTVVVDNSVLEIYANGRFALSTWARSWYKNSTDIKFYHNGAGEV
XP_003190558.1 541 RLFDLQGDAQEIETLDLTVVVDNSVLEIYANGRFALSTWARSWYKNSTDIKFFHNGAGEV
ABW87267.1 541 RLFDLQGDAQEIETLDLTVVVDNSVLEIYANGRFALSTWARYVFCDGR-----QQGIG--
consensus 541 ********.********************************..............*.*..


EIT76425.1 601 TFSNVTVSEGLFEAWPERV
XP_002383662.1 601 TFSNVTVSEGLFEAWPERV
BfrA 601 TFSNVTVSEGLFEAWPERV
XP_003190558.1 601 TFSNVTVSEGLFEAWPERV
ABW87267.1 -------------------
consensus 601 ...................
