# Supplementary figures and images for: Purification, Cloning, Characterization, and N-Glycosylation Analysis of a Novel β-Fructosidase from Aspergillus oryzae FS4 Synthesizing Levan- and Neolevan-Type Fructooligosaccharides
Source: PLoS One. 2014 Dec 12;9(12):e114793. doi: 10.1371/journal.pone.0114793 (PMC4264766; doi:10.1371/journal.pone.0114793)

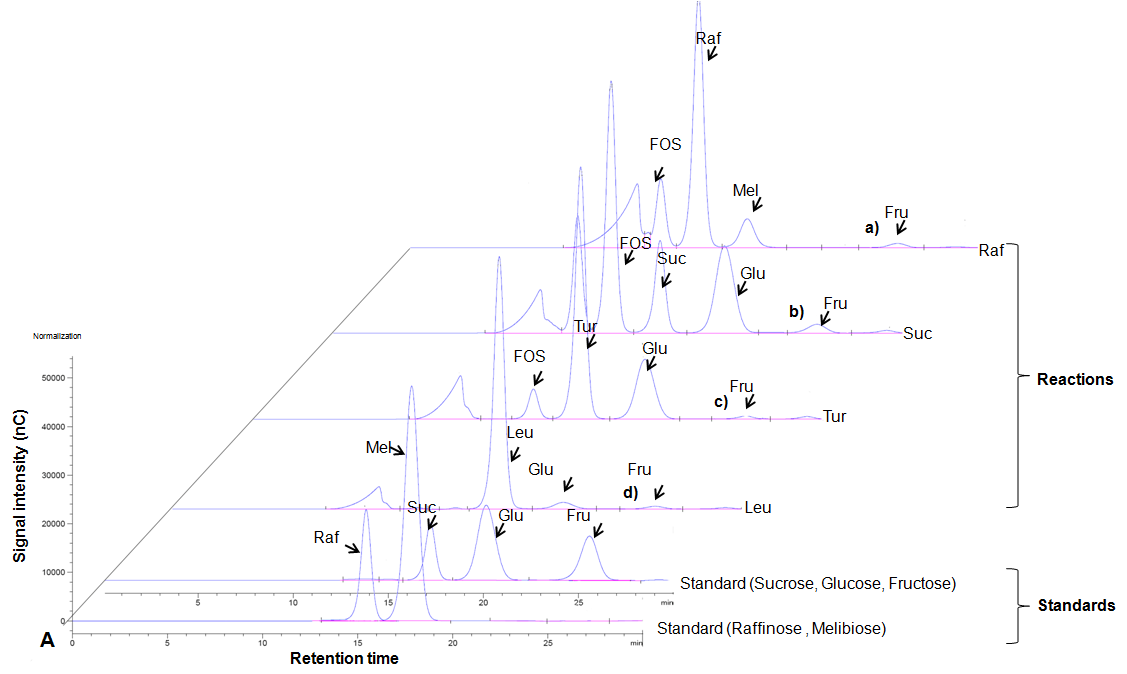


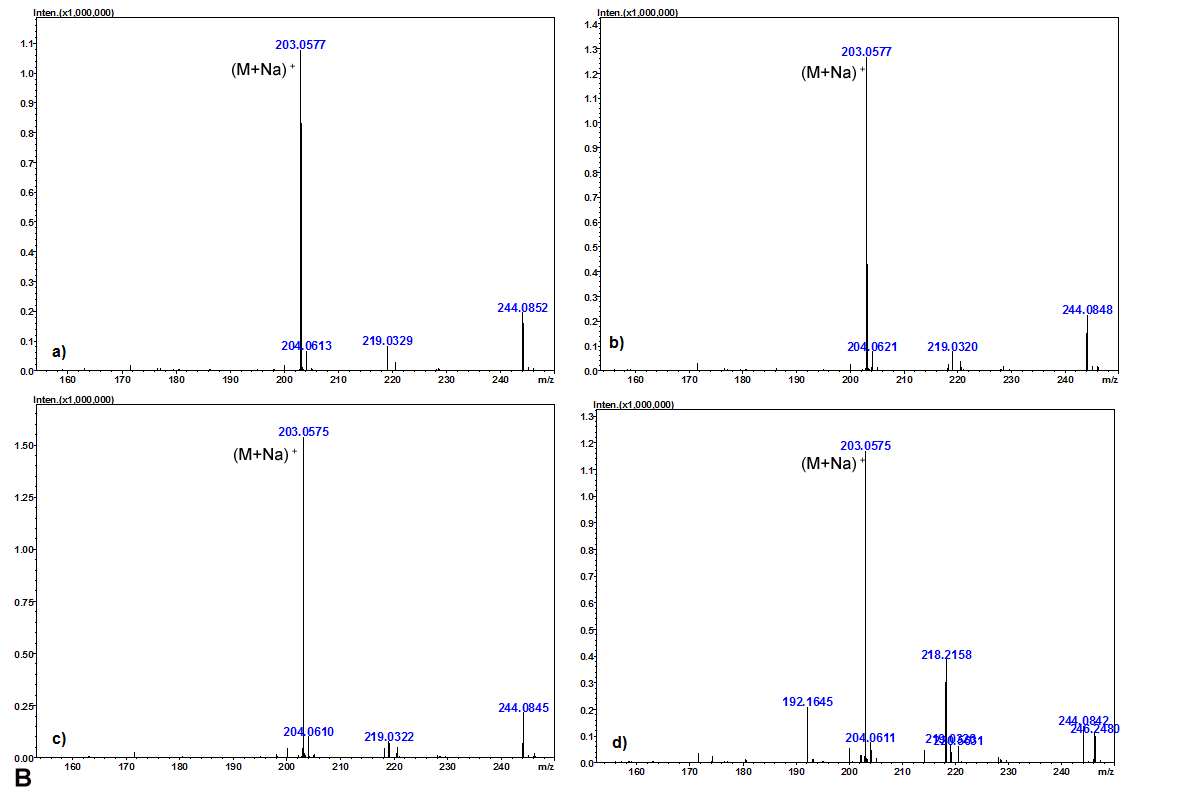

Supplement: S3 Figure — Hydrolytic substrate specificity analysis. A: The chromatogram of the reaction products by HPLC. The reactions were performed used N.BfrA (36 ng) incubated with 1 M substrates [sucrose (Suc), raffinose (Raf), turanose (Tur), and leucrose(Leu)] for 10 min at 55°C. The reactions were analyzed by HPLC through an Aminex HPX-42 column (300×7.8 mm, Bio-Rad, USA). The peaks corresponding to sucrose (Suc), glucose (Glc), fructose (Frc), raffinose (Raf), turanose (Tur), leucrose (Leu), and FOS were indicated. B: Mass spectra of hydrolysis products (fructose) of reactions. The fractions a), b), c), and d) in A were collected and analyzed by LC/MS-IT-TOF (Shimadzu, Japan) in positive ion mode. (DOCX) [file pone.0114793.s003.docx]

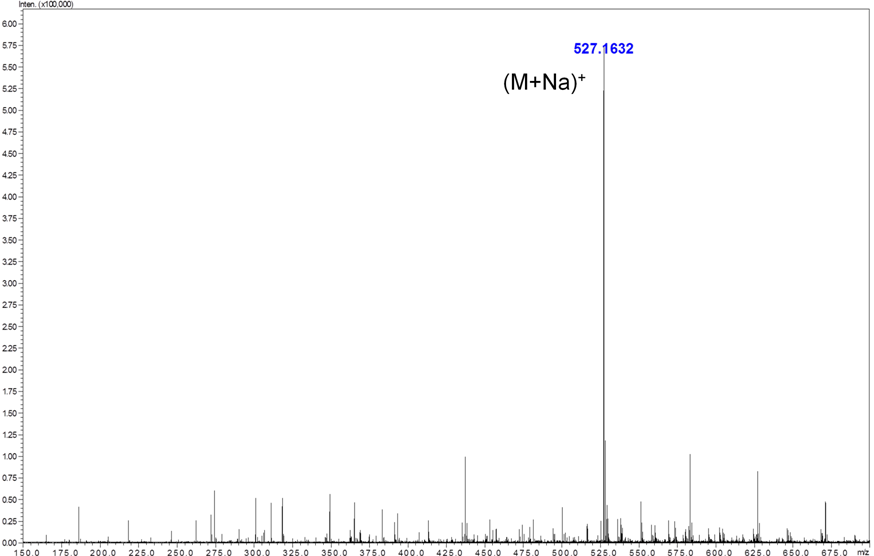

Supplement: S5 Figure — Mass data of compound identified as neokestose. (DOC) [file pone.0114793.s005.doc]

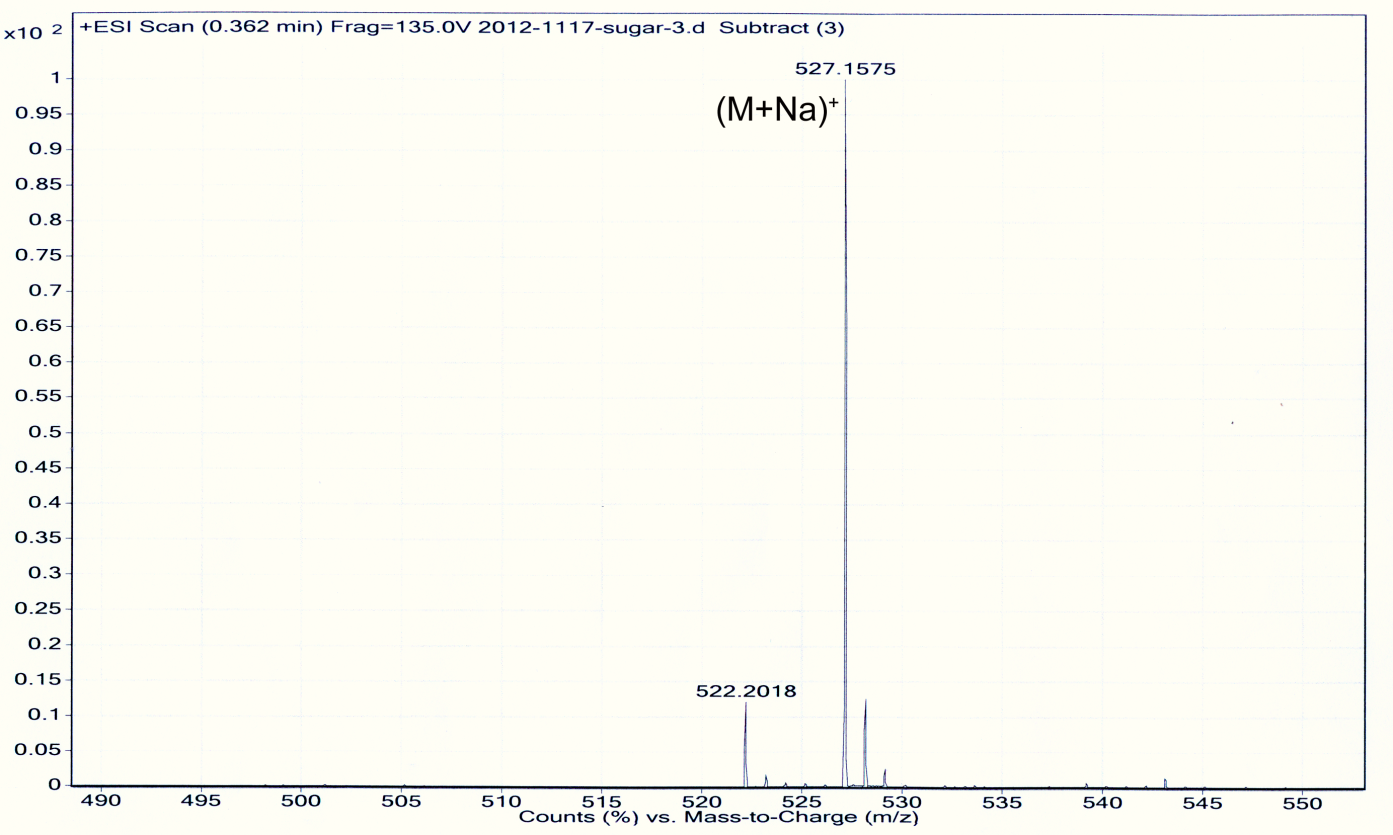

Supplement: S6 Figure — Mass data of compound identified as 6-kestose. (DOCX) [file pone.0114793.s006.docx]

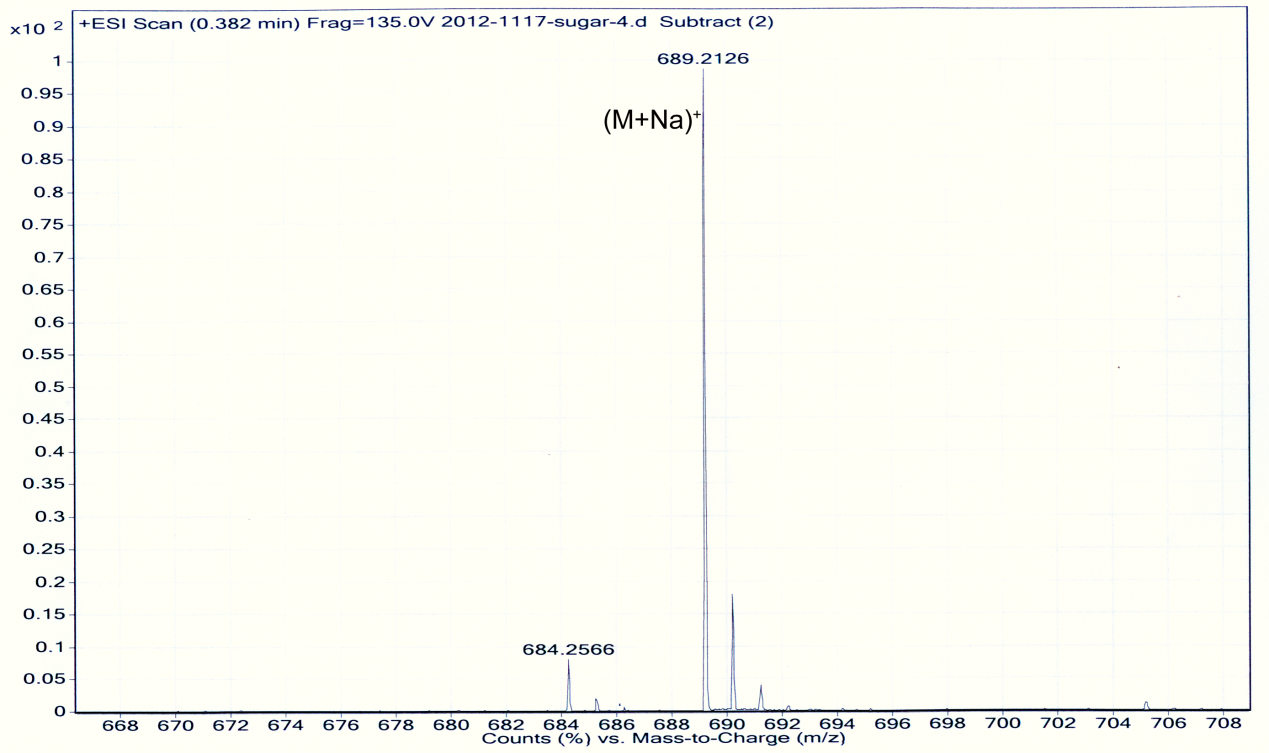

Supplement: S7 Figure — Mass data of compound identified as and 6-nystose. (DOCX) [file pone.0114793.s007.docx]

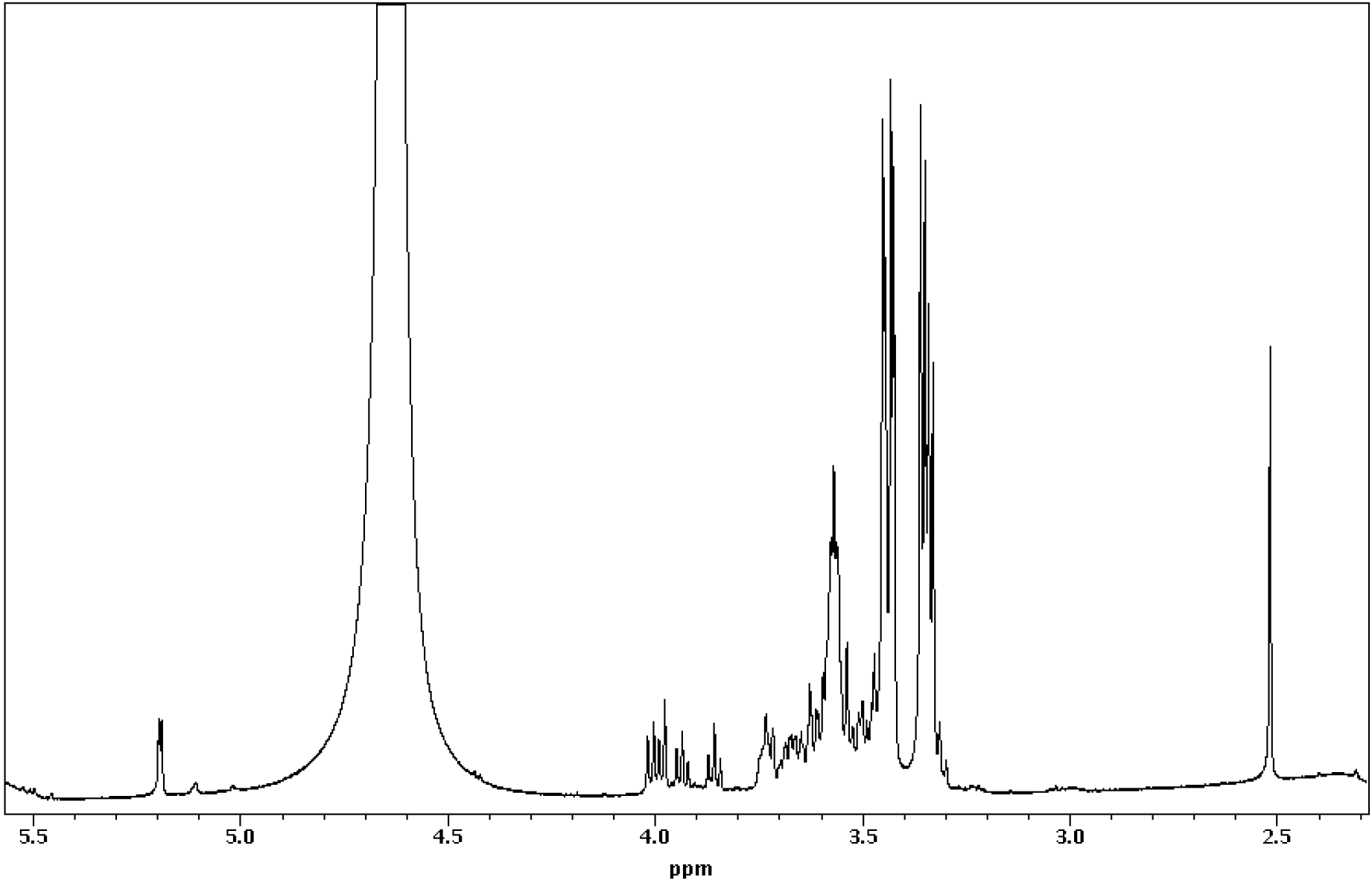

Supplement: S8 Figure — 1H NMR spectrum of neokestose. (TIF) [file pone.0114793.s008.tif]

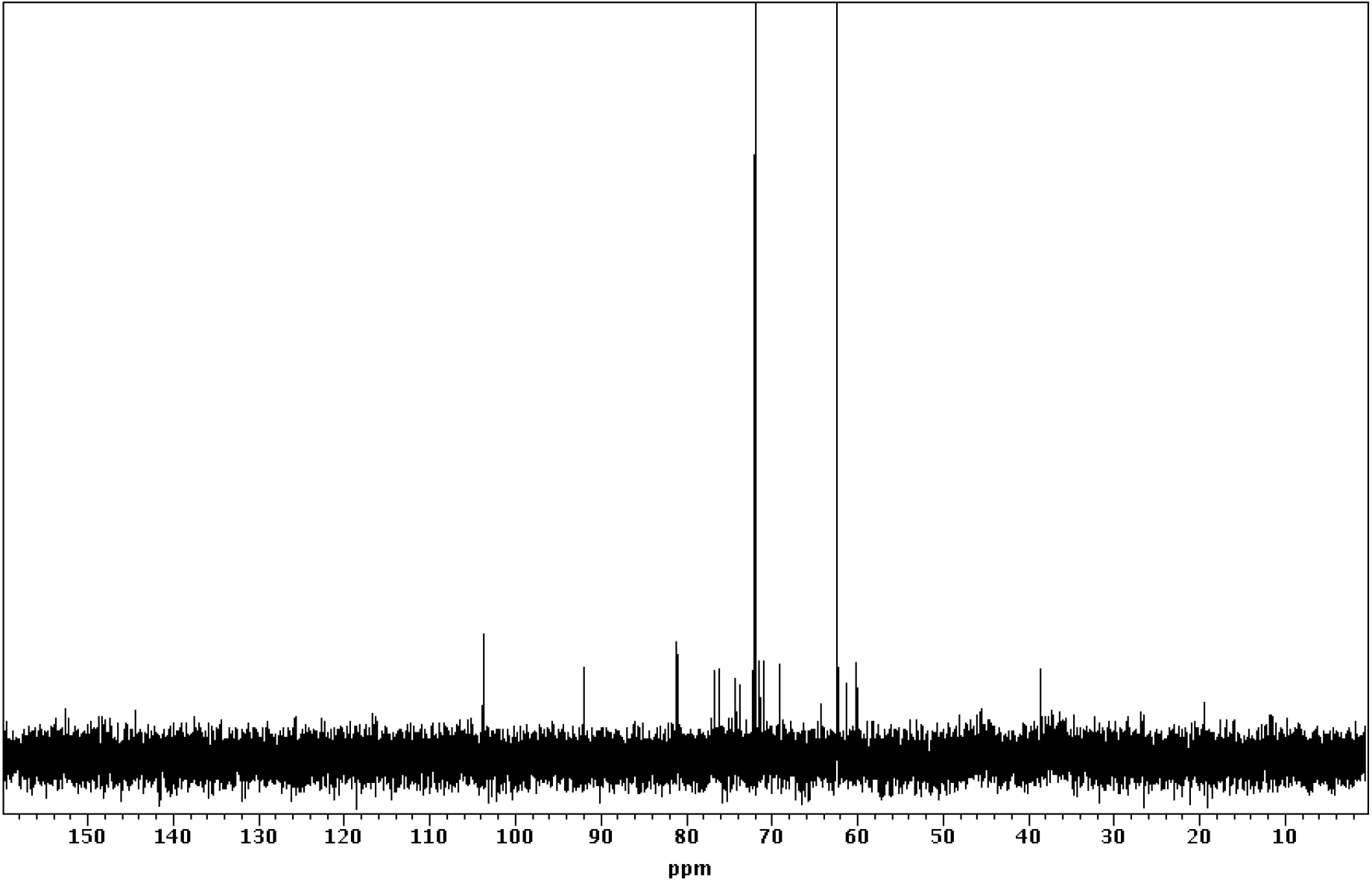

Supplement: S9 Figure — 13C NMR spectrum of neokestose. (TIF) [file pone.0114793.s009.tif]

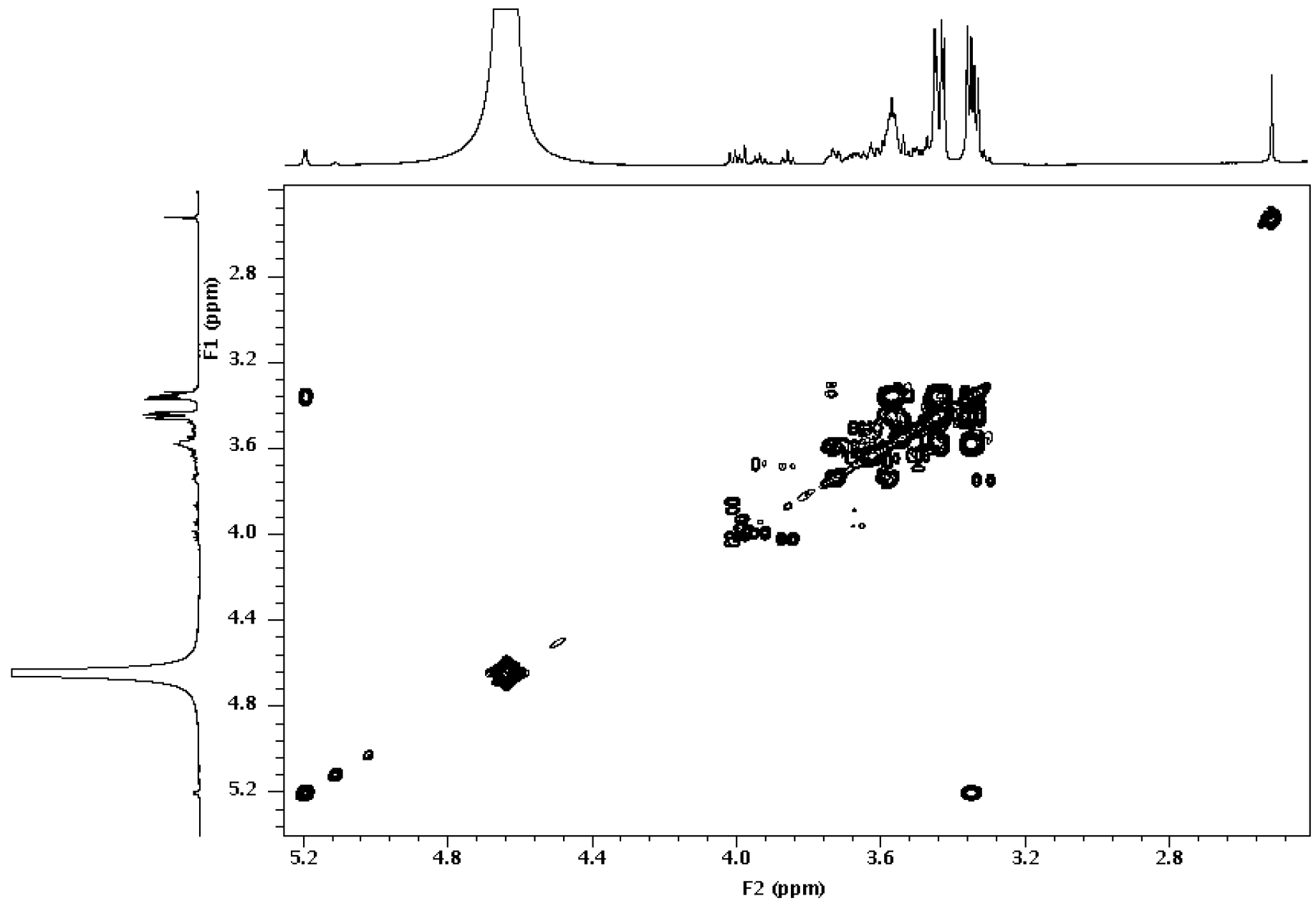

Supplement: S10 Figure — COSY spectrum of neokestose. (TIF) [file pone.0114793.s010.tif]

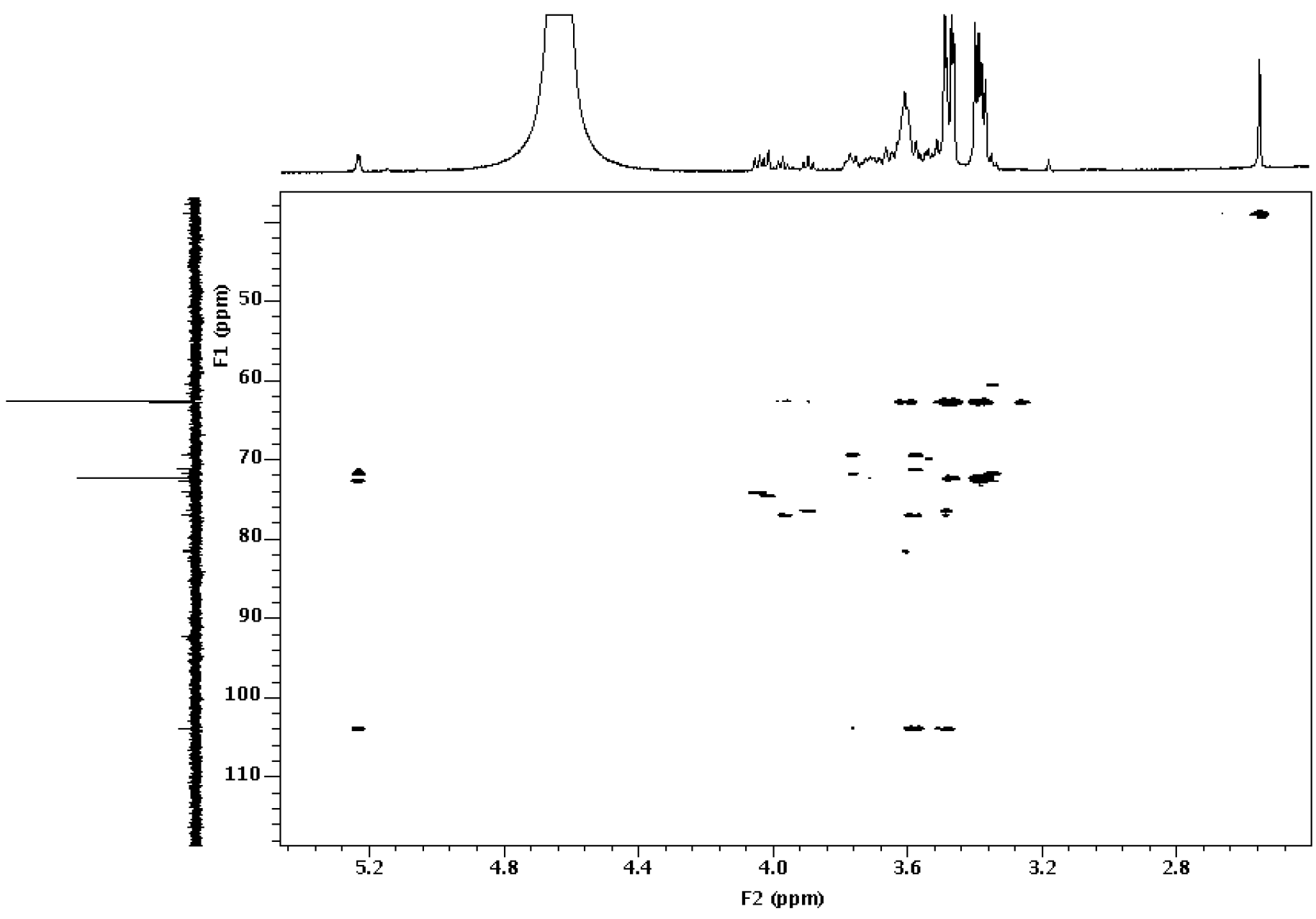

Supplement: S11 Figure — HMBC spectrum of neokestose. (TIF) [file pone.0114793.s011.tif]

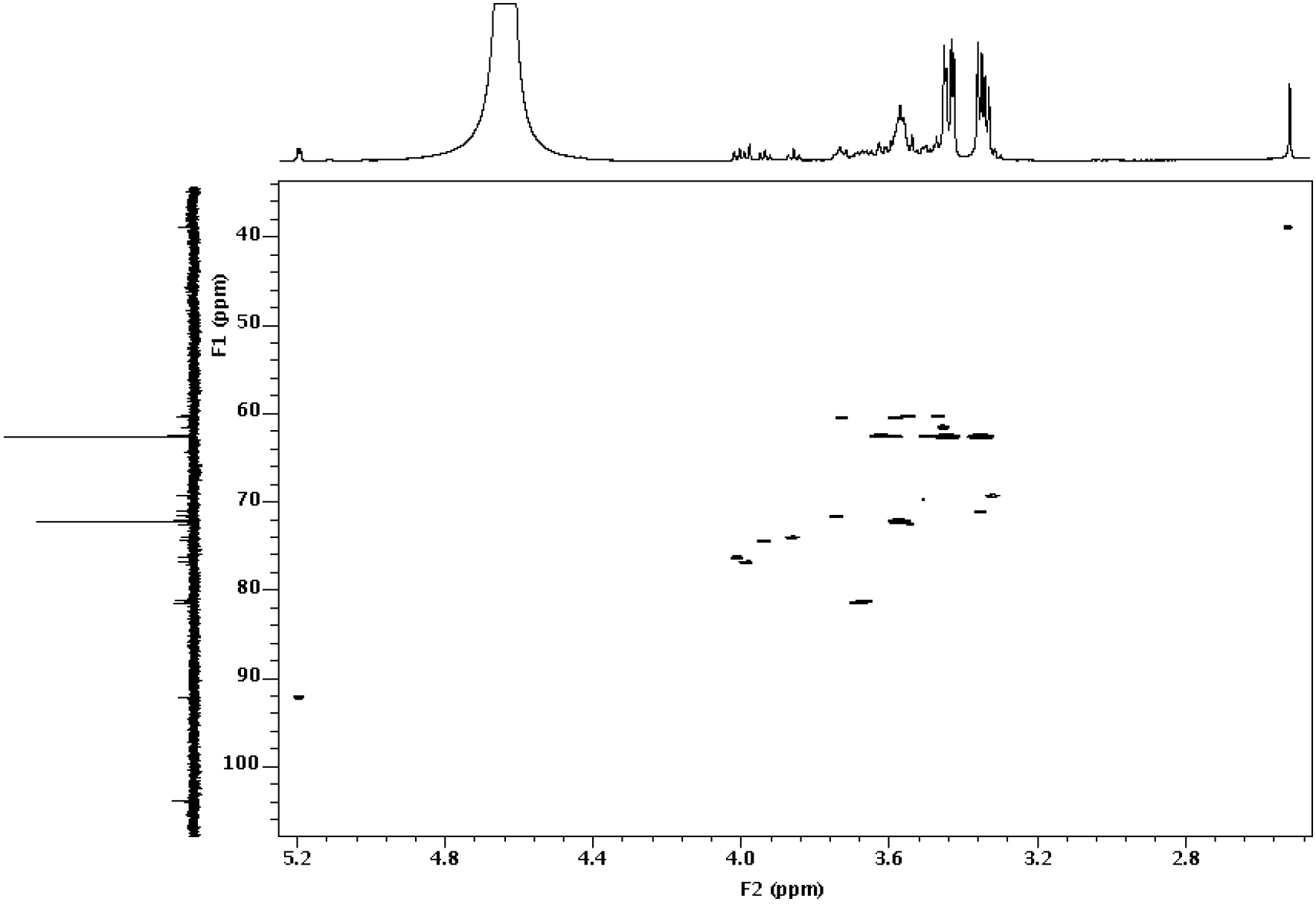

Supplement: S12 Figure — HSQC spectrum of neokestose. (TIF) [file pone.0114793.s012.tif]

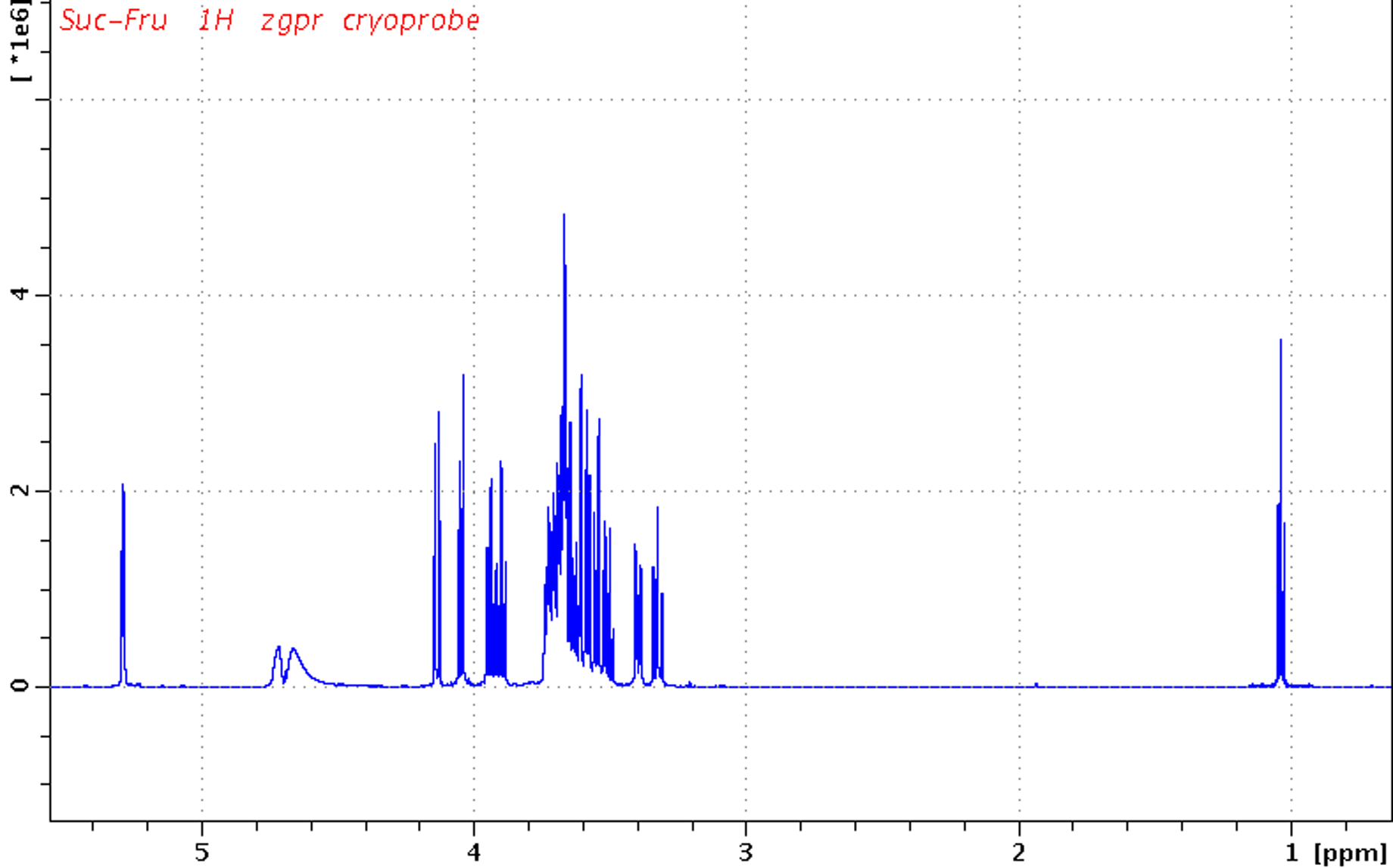

Supplement: S13 Figure — 1H NMR spectrum of 6-kesose (TIF) [file pone.0114793.s013.tif]

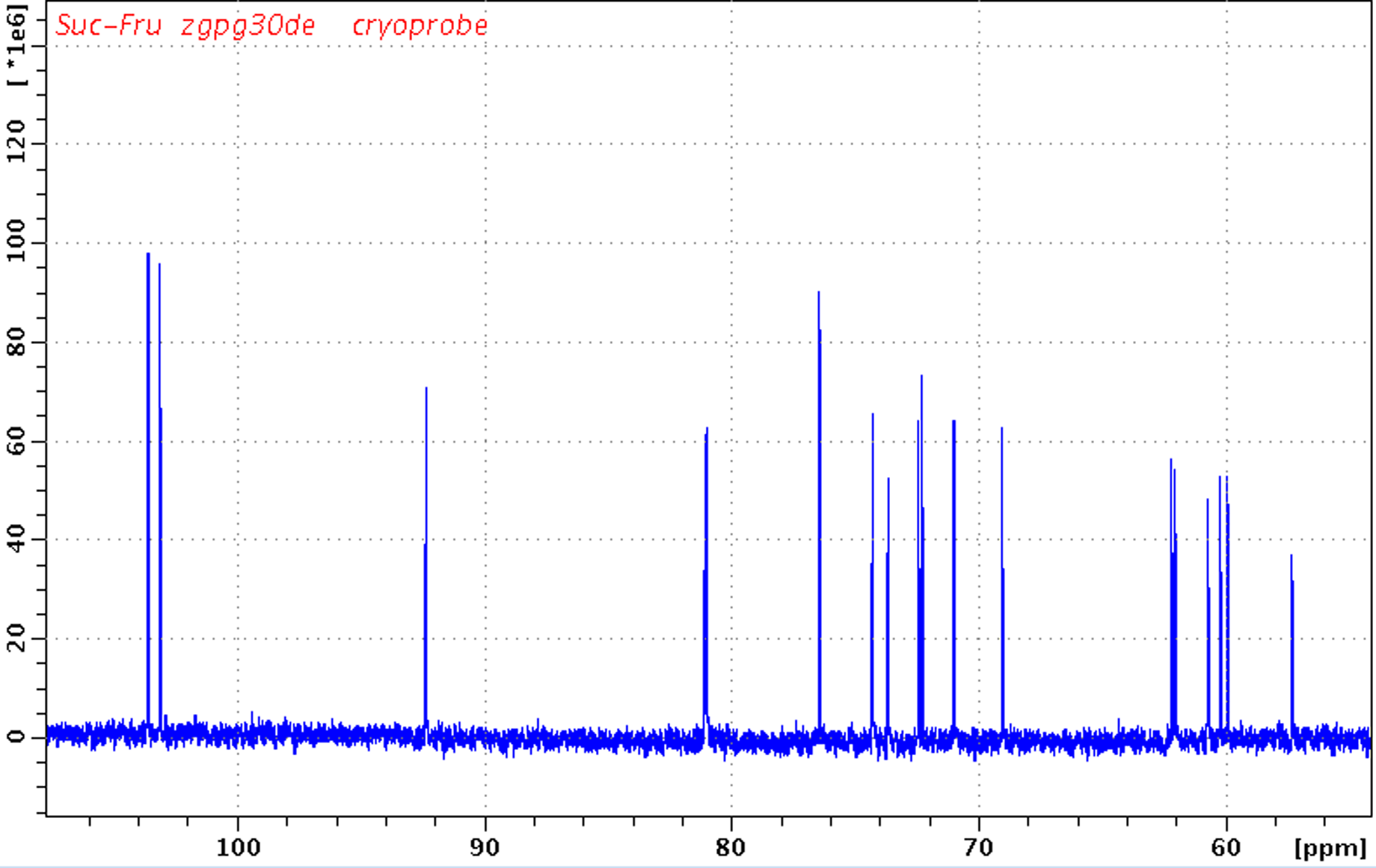

Supplement: S14 Figure — 13C NMR spectrum of 6-kesose. (TIF) [file pone.0114793.s014.tif]

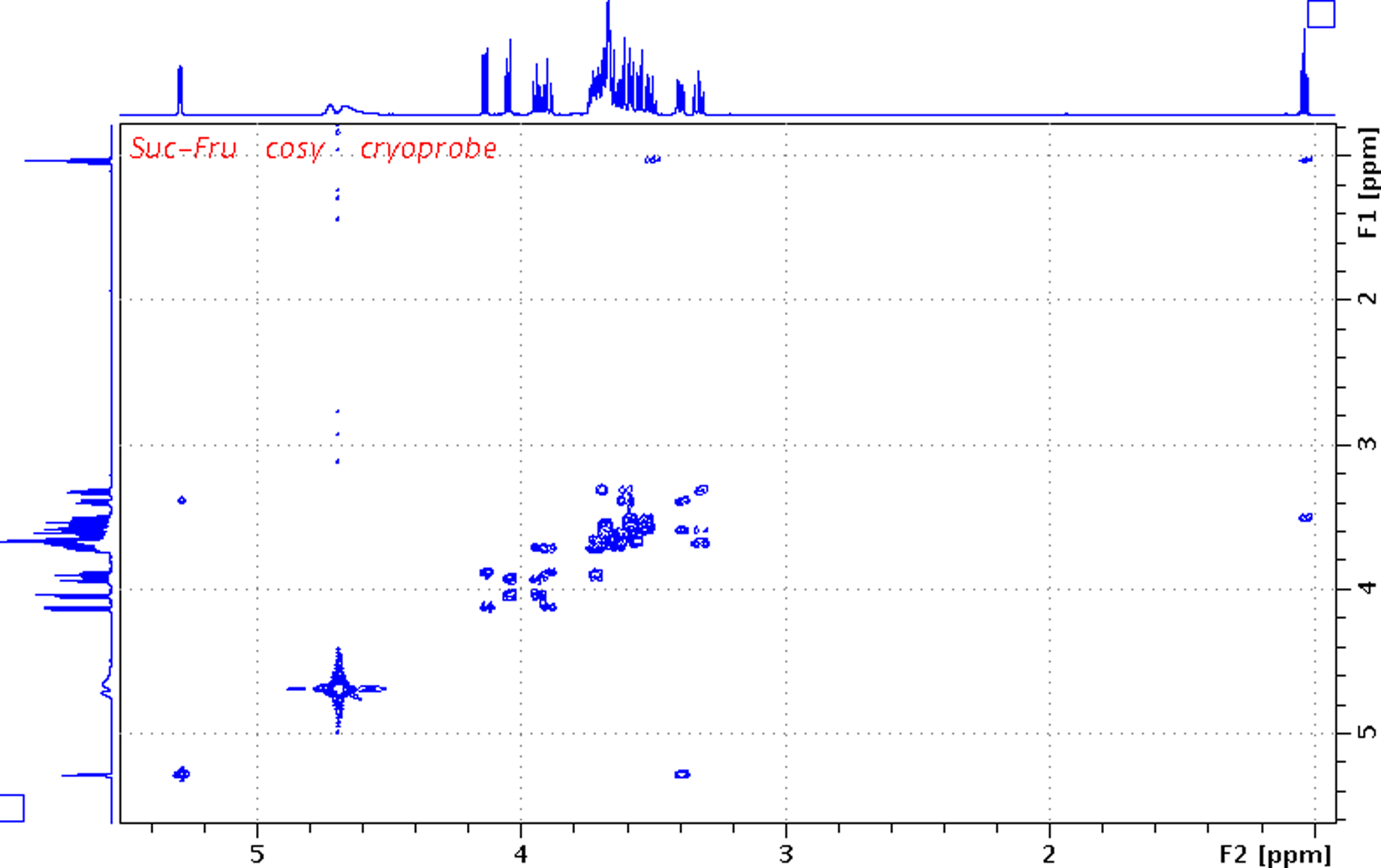

Supplement: S15 Figure — COSY spectrum of 6-kesose. (TIF) [file pone.0114793.s015.tif]

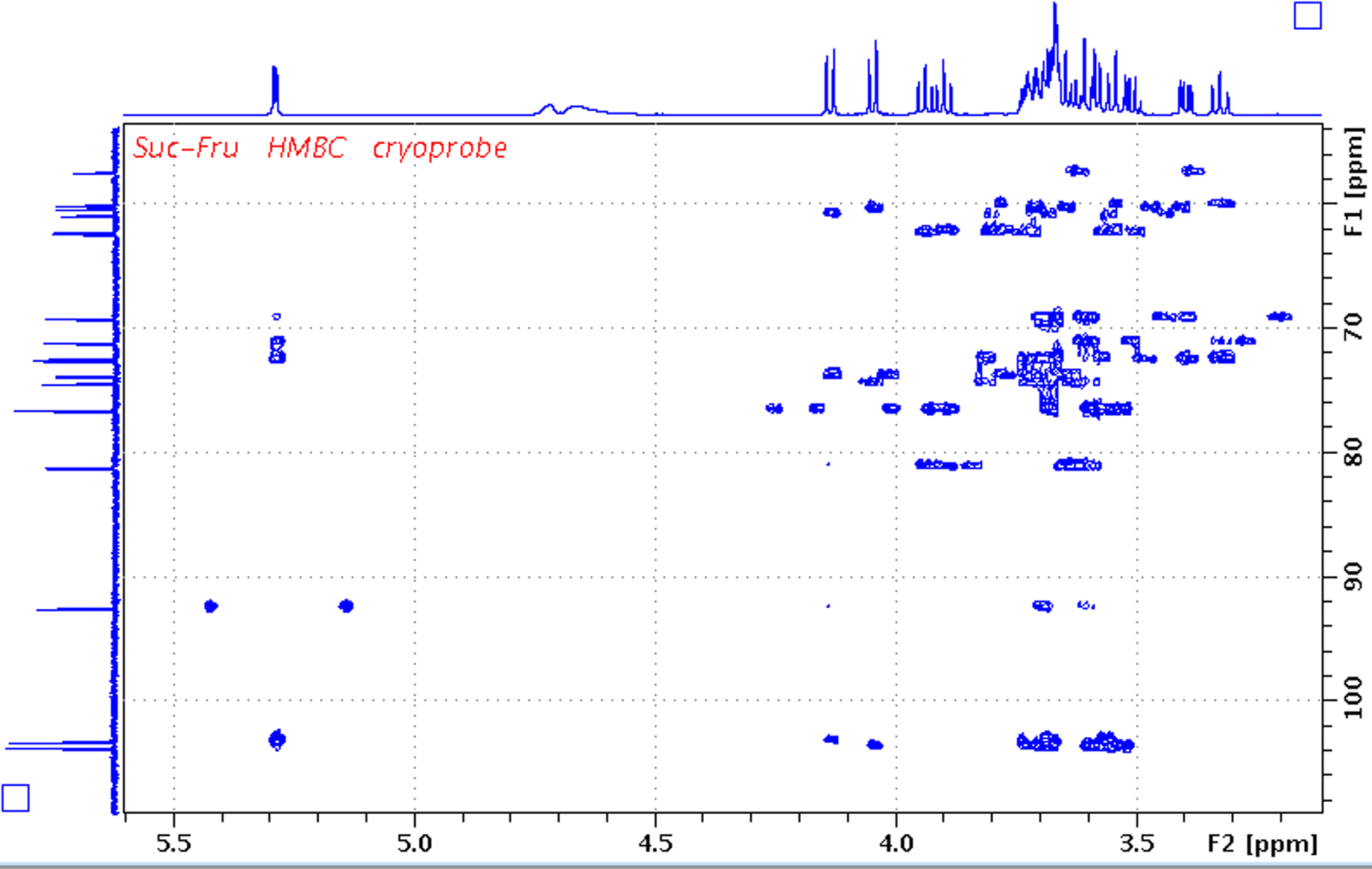

Supplement: S16 Figure — HMBC spectrum of 6-kesose. (TIF) [file pone.0114793.s016.tif]

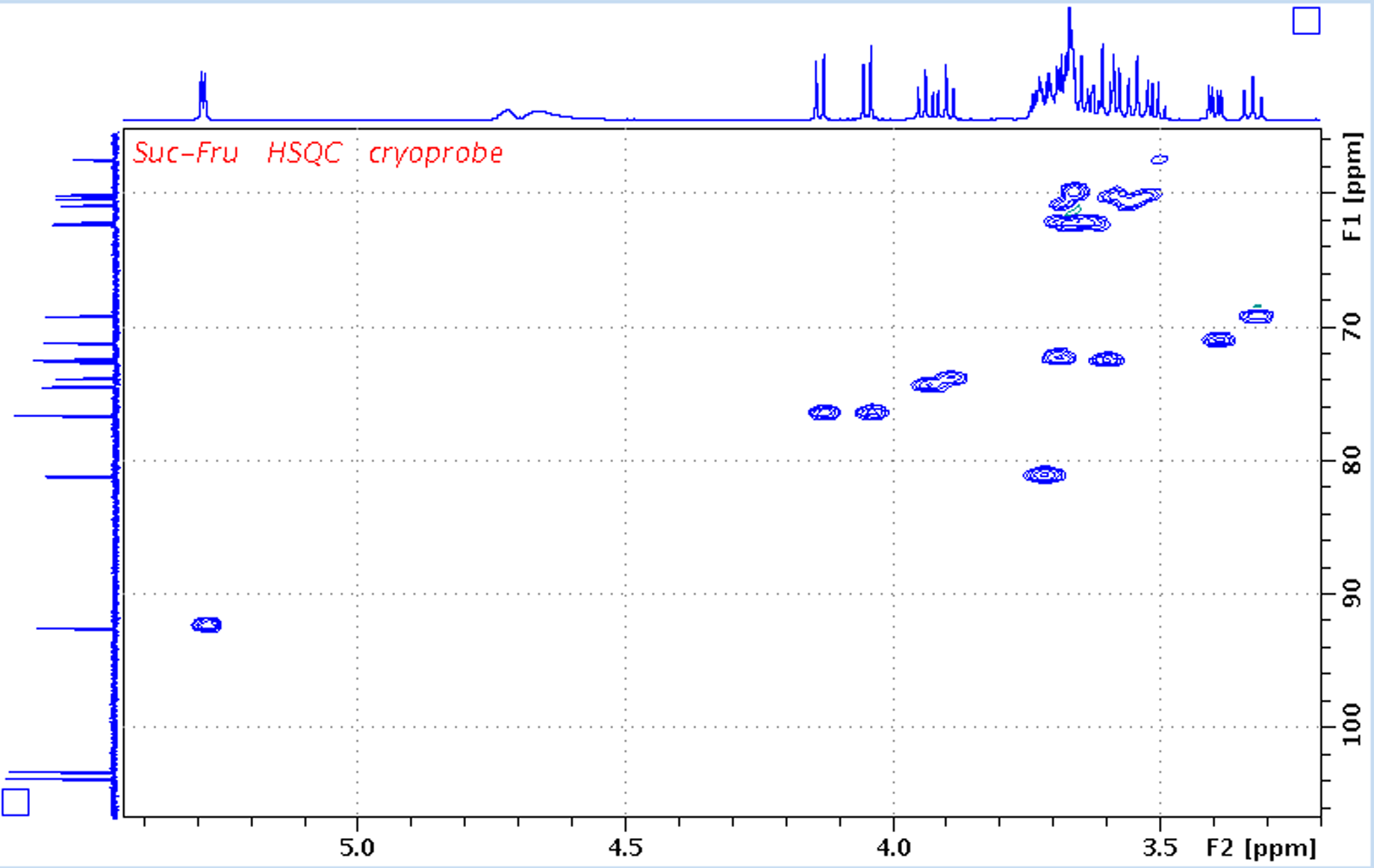

Supplement: S17 Figure — HSQC spectrum of 6-kesose. (TIF) [file pone.0114793.s017.tif]

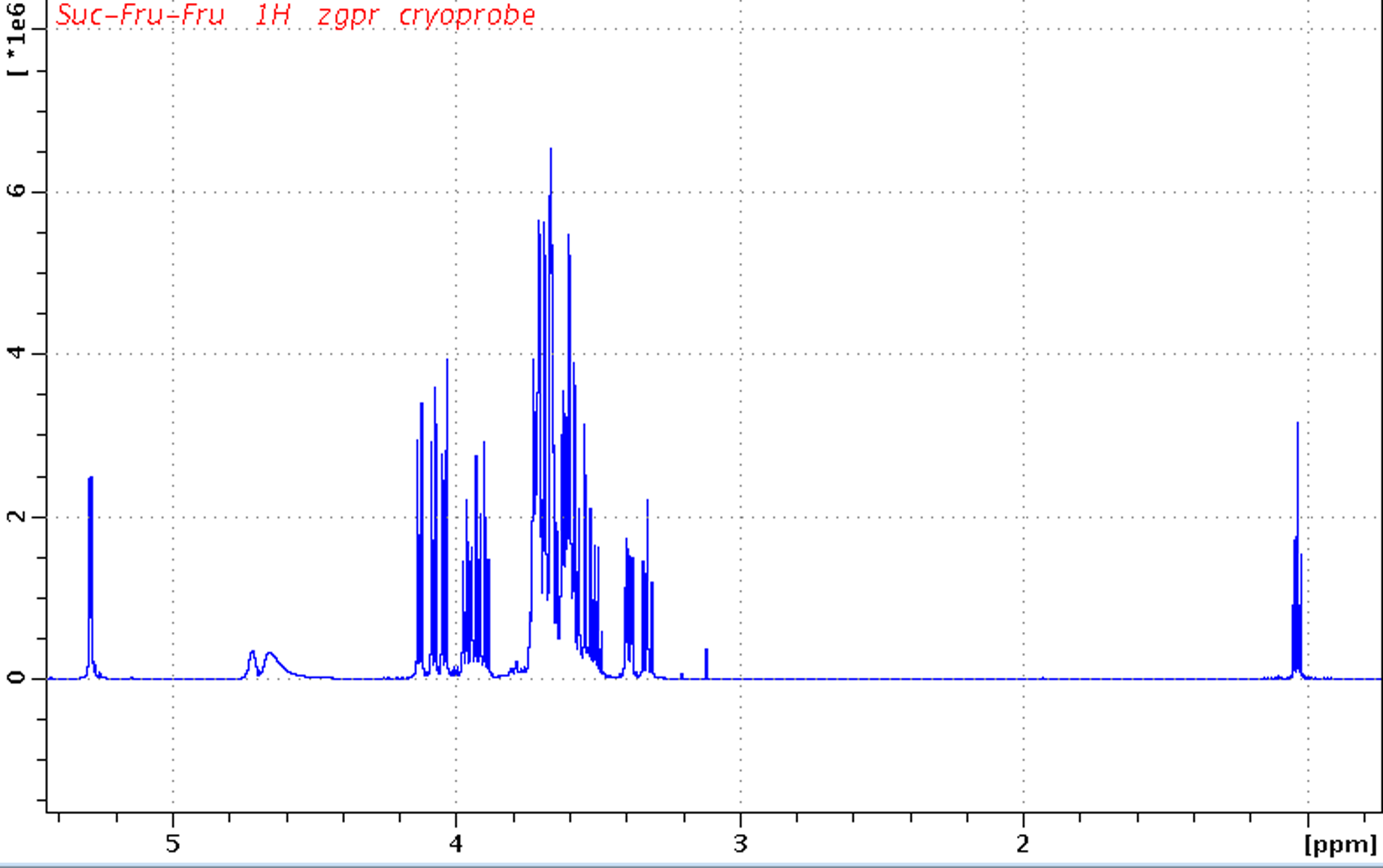

Supplement: S18 Figure — 1H NMR spectrum of 6-nystose. (TIF) [file pone.0114793.s018.tif]

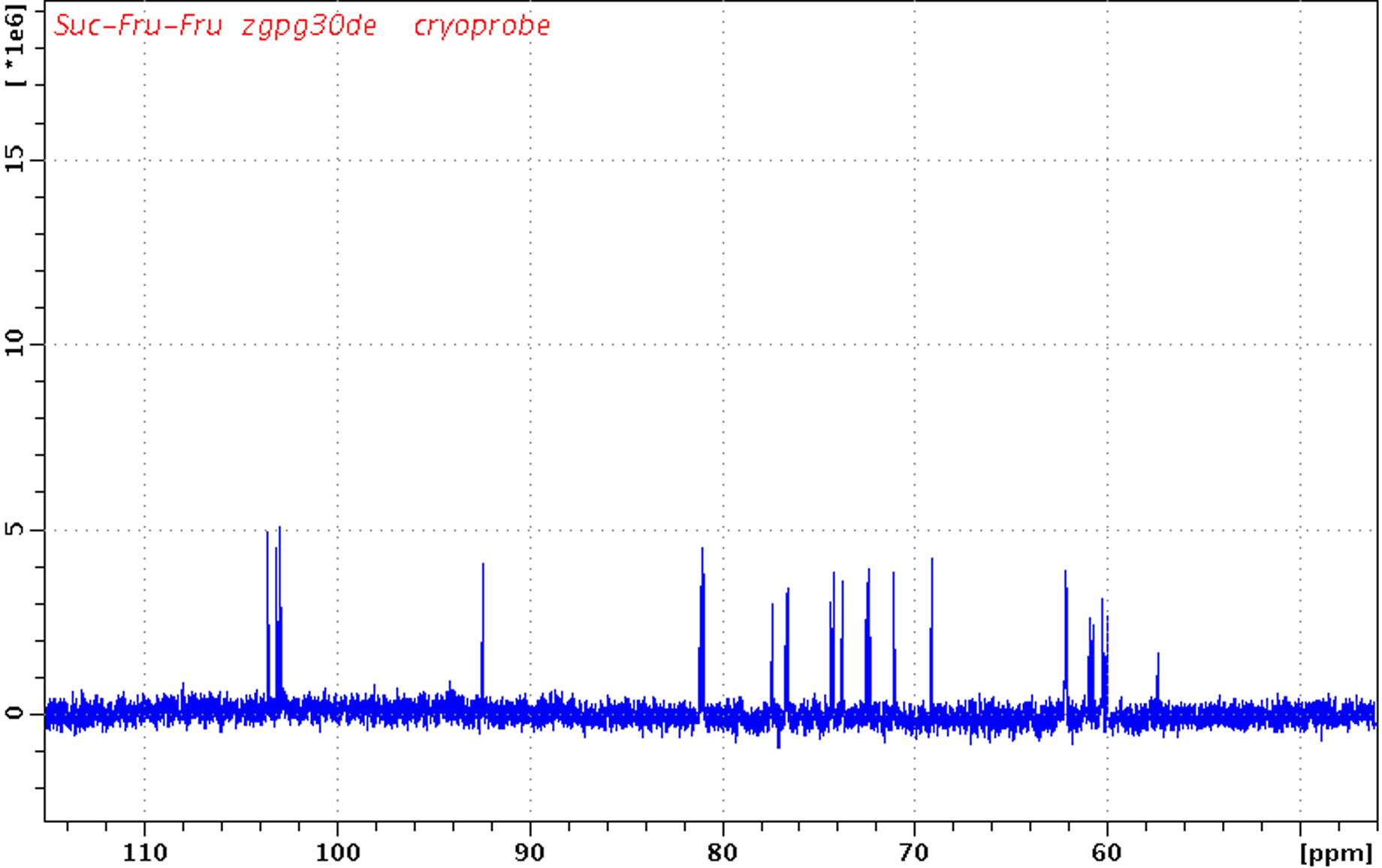

Supplement: S19 Figure — 13C NMR spectrum of 6-nystose. (TIF) [file pone.0114793.s019.tif]

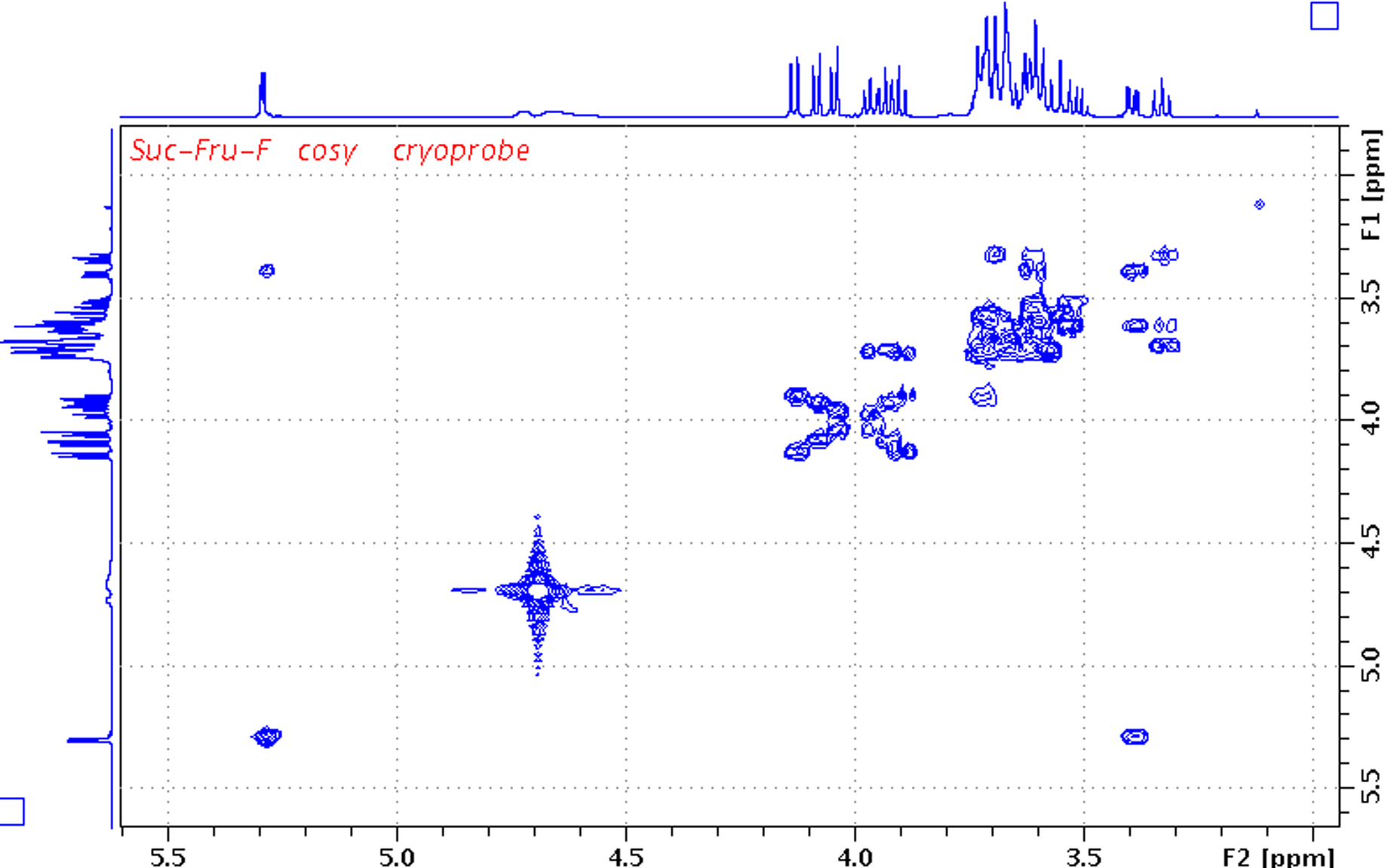

Supplement: S20 Figure — COSY spectrum of 6-nystose. (TIF) [file pone.0114793.s020.tif]

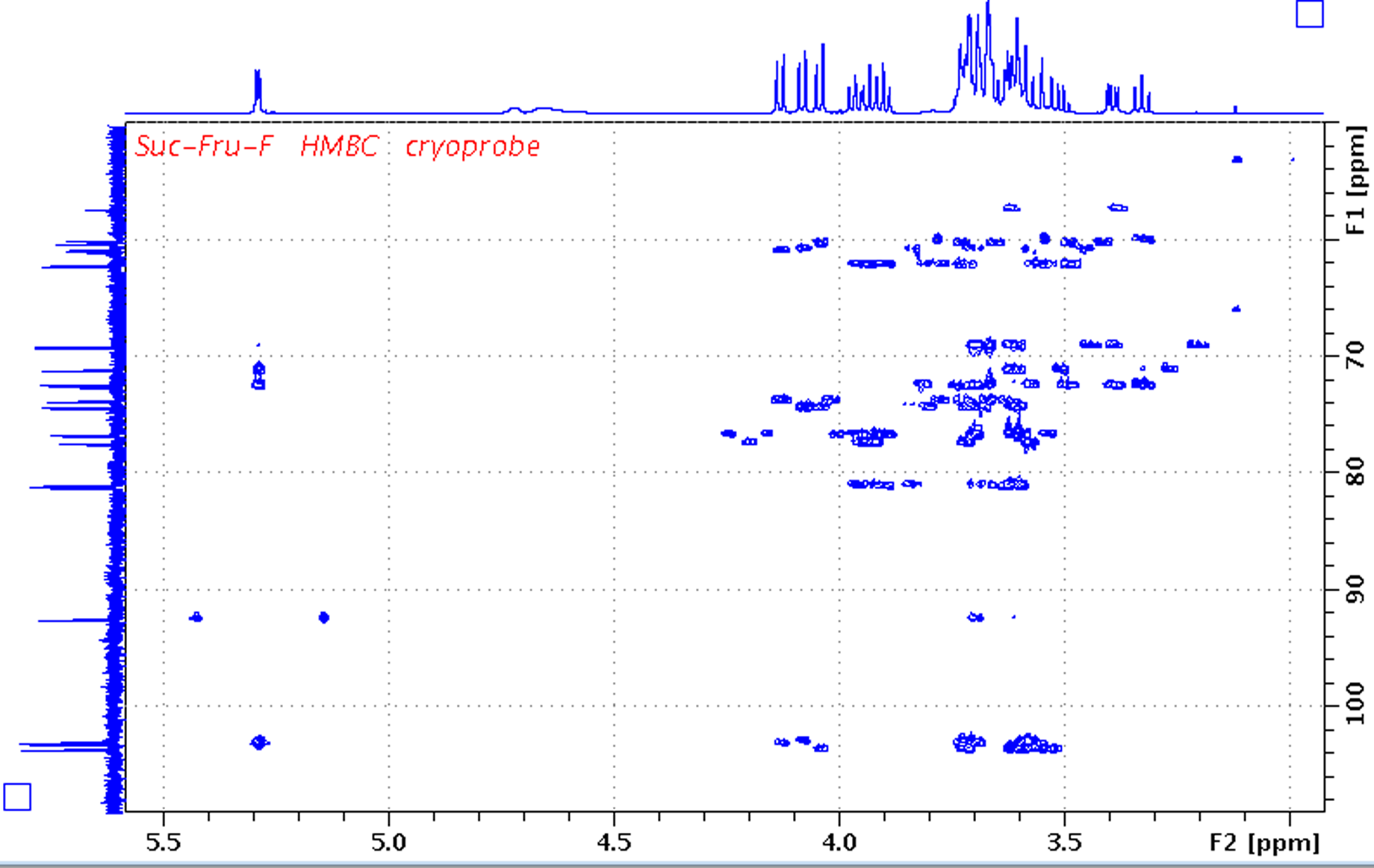

Supplement: S21 Figure — HMBC spectrum of 6-nystose. (TIF) [file pone.0114793.s021.tif]

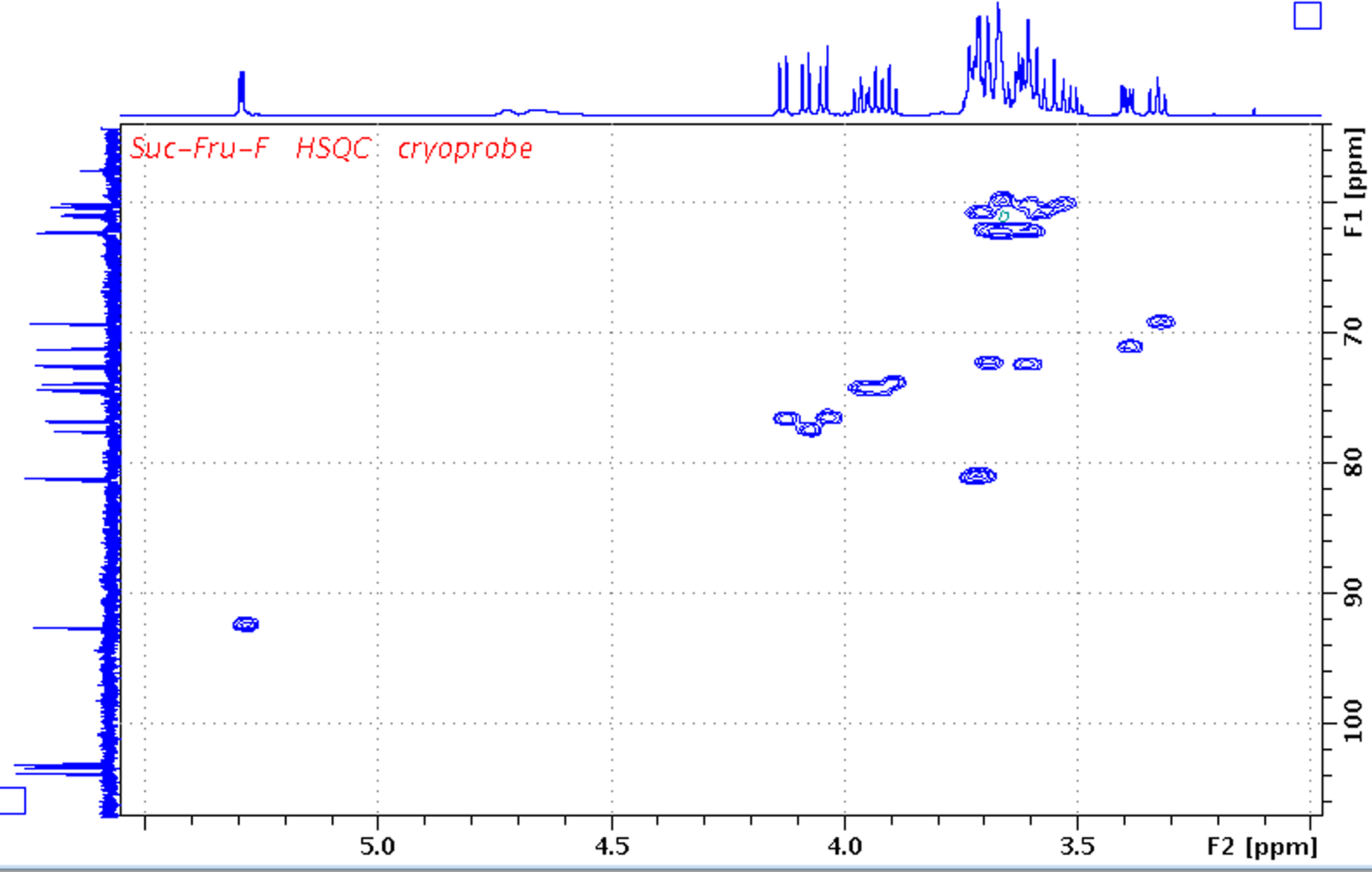

Supplement: S22 Figure — HSQC spectrum of 6-nystose. (TIF) [file pone.0114793.s022.tif]
