# Supplementary material for: Purification, Cloning, Characterization, and N-Glycosylation Analysis of a Novel β-Fructosidase from Aspergillus oryzae FS4 Synthesizing Levan- and Neolevan-Type Fructooligosaccharides
Source: PLoS One. 2014 Dec 12;9(12):e114793. doi: 10.1371/journal.pone.0114793 (PMC4264766; doi:10.1371/journal.pone.0114793)
Supplement: Supplementary file 3 [file pone.0114793.s001.docx]

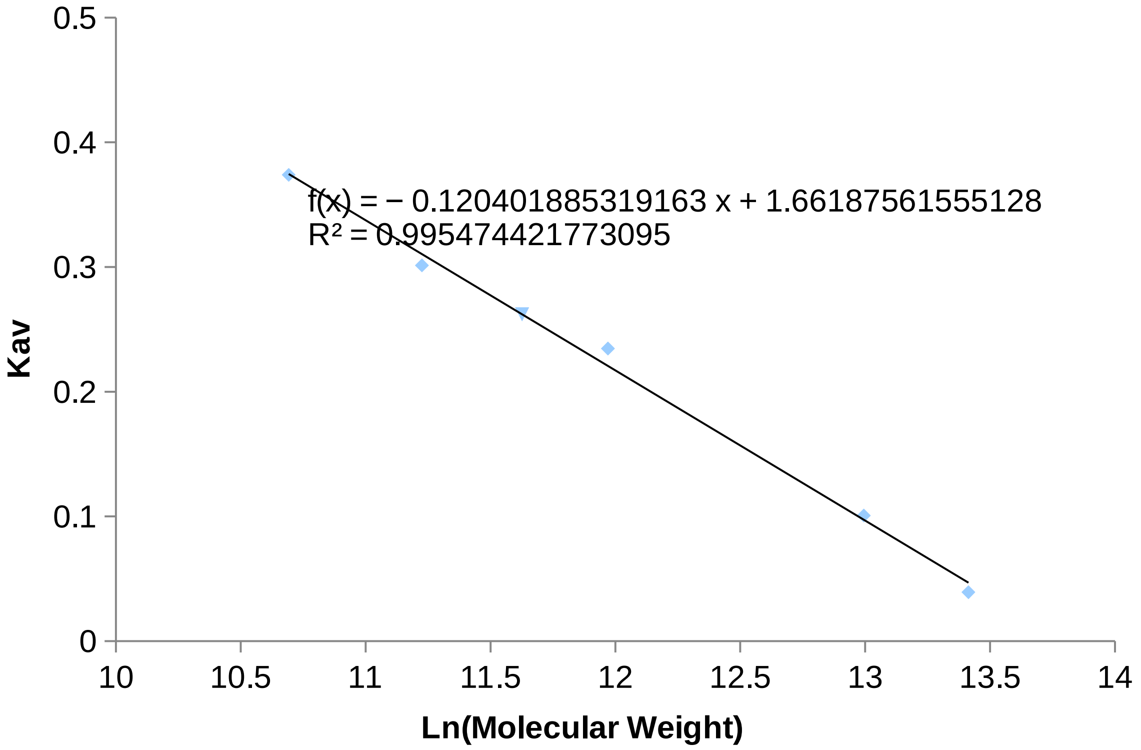

f(x) = − 0.120401885319163 x + 1.66187561555128
R² = 0.995474421773095
Kav
Ln(Molecular Weight)
0.5
0.4
0.3
0.2
0.1
0
10
10.5
11
11.5
12
12.5
13
13.5
14
