# Supplementary material for: Purification, Cloning, Characterization, and N-Glycosylation Analysis of a Novel β-Fructosidase from Aspergillus oryzae FS4 Synthesizing Levan- and Neolevan-Type Fructooligosaccharides
Source: PLoS One. 2014 Dec 12;9(12):e114793. doi: 10.1371/journal.pone.0114793 (PMC4264766; doi:10.1371/journal.pone.0114793)
Supplement: Supplementary file 4 [file pone.0114793.s004.docx]

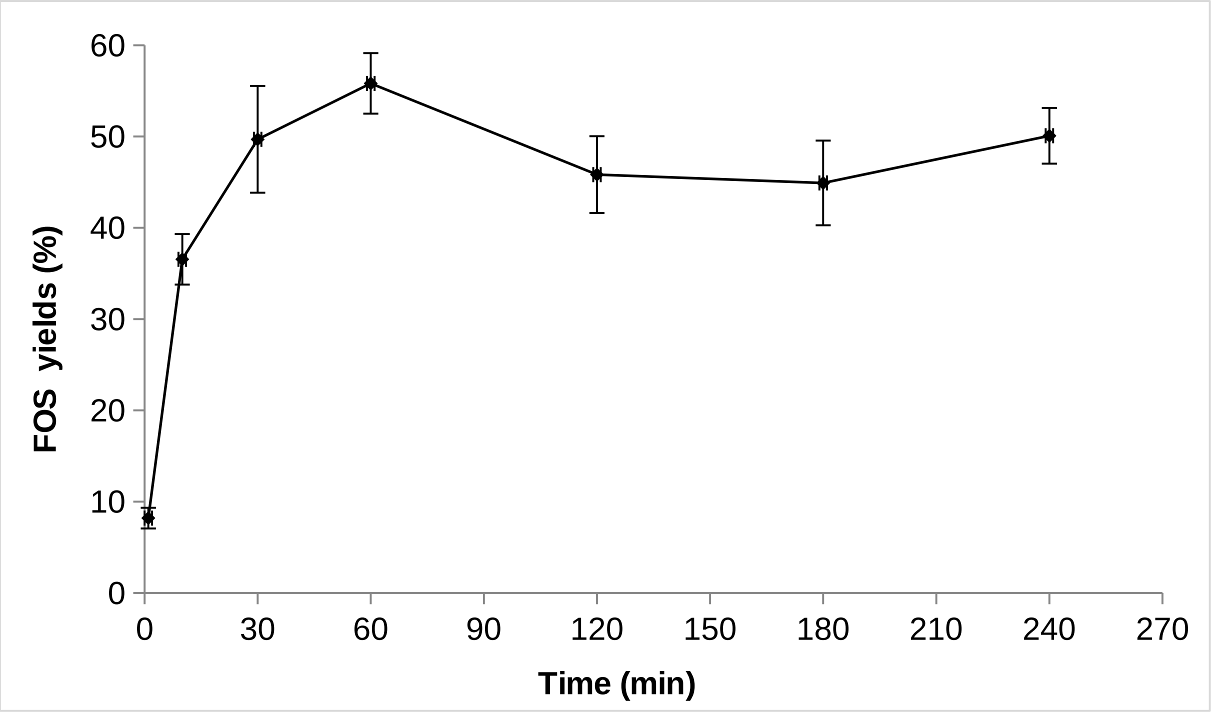

60
50
40
30
20
10
0
FOS yields (%)
0
30
60
90
120
150
180
210
240
270
Time (min)
